# Antioxidant supplements promote tumor formation and growth and confer drug resistance in hepatocellular carcinoma by reducing intracellular ROS and induction of TMBIM1

**DOI:** 10.1186/s13578-021-00731-0

**Published:** 2021-12-19

**Authors:** Vanilla Xin Zhang, Karen Man-Fong Sze, Lo-Kong Chan, Daniel Wai-Hung Ho, Yu-Man Tsui, Yung-Tuen Chiu, Eva Lee, Abdullah Husain, Hongyang Huang, Lu Tian, Carmen Chak-Lui Wong, Irene Oi-Lin Ng

**Affiliations:** 1 Department of Pathology, The University of Hong Kong, Pokfulam, Hong Kong SAR; 2 State Key Laboratory of Liver Research, The University of Hong Kong, Pokfulam, Hong Kong SAR

**Keywords:** Hepatocellular carcinoma, Antioxidants, *N*-acetylcysteine, Glutathione, Sorafenib, Reactive oxygen species, NRF2, TMBIM1

## Abstract

**Background:**

Controversy over the benefits of antioxidants supplements in cancers persists for long. Using hepatocellular carcinoma (HCC) as a model, we investigated the effects of exogenous antioxidants *N*-acetylcysteine (NAC) and glutathione (GSH) on tumor formation and growth.

**Methods:**

Multiple mouse models, including diethylnitrosamine (DEN)-induced and Trp53KO/C-MycOE-induced HCC models, mouse hepatoma cell and human HCC cell xenograft models with subcutaneous or orthotopic injection were used. In vitro assays including ROS assay, colony formation, sphere formation, proliferation, migration and invasion, apoptosis, cell cycle assays were conducted. Western blot was performed for protein expression and RNA-sequencing to identify potential gene targets.

**Results:**

In these multiple different mouse and cell line models, we observed that NAC and GSH promoted HCC tumor formation and growth, accompanied with significant reduction of intracellular reactive oxygen species (ROS) levels. Moreover, NAC and GSH promoted cancer stemness, and abrogated the tumor-suppressive effects of Sorafenib both in vitro and in vivo. Exogenous supplementation of NAC or GSH reduced the expression of NRF2 and GCLC, suggesting the NRF2/GCLC-related antioxidant production pathway might be desensitized. Using transcriptomic analysis to identify potential gene targets, we found that TMBIM1 was significantly upregulated upon NAC and GSH treatment. Both TCGA and in-house RNA-sequence databases showed that TMBIM1 was overexpressed in HCC tumors. Stable knockdown of TMBIM1 increased the intracellular ROS; it also abolished the promoting effects of the antioxidants in HCC cells. On the other hand, BSO and SSA, inhibitors targeting NAC and GSH metabolism respectively, partially abrogated the pro-oncogenic effects induced by NAC and GSH in vitro and in vivo.

**Conclusions:**

Our data implicate that exogenous antioxidants NAC and GSH, by reducing the intracellular ROS levels and inducing TMBIM expression, promoted HCC formation and tumor growth, and counteracted the therapeutic effect of Sorafenib. Our study provides scientific insight regarding the use of exogenous antioxidant supplements in cancers.

**Supplementary Information:**

The online version contains supplementary material available at 10.1186/s13578-021-00731-0.

## Background

Antioxidant supplements are popular health boosters and some of them are claimed to possess anti-cancer activity. There is a long-standing debate over the benefits of antioxidants supplements intake not only in healthy individuals but also in patients diagnosed with cancers [1]. In normal physiological conditions, various cellular activities generate reactive oxygen species (ROS), and the presence of counteractive antioxidant molecules serve as electron donors to neutralize and inhibit the accumulation of ROS, ensuring the integrity of the genome and other cellular protein structures. In cancer cells, the increased metabolic activities underlying the abnormally high proliferation rate unavoidably increase the cellular ROS production. To withstand the potential cytotoxic effects brought by this unexceptionally high level of ROS, cancer cells are capable to regulate ROS levels by modulating the cellular antioxidant production, which allows them to adapt to oxidative stress and thus ensures their sustainable propagation [2].

Some previous studies have shown that antioxidant supplements may exert anti-tumor effects. For example, Vitamin C could exhibit selective anti-tumor effects alone or when combined with other therapies [3, 4] while Vitamin D could reduce drug resistance in certain solid and non-solid cancers [5]. Also, Vitamin E could prevent breast cancer metastasis by lowering programmed cell death protein 1 (PD-L1) expression in the presence of interferon-γ [6]. On the other hand, some large-scale, randomized clinical trials yielded mixed and controversial results, which provide us with some insight into the adverse effects regarding the use of antioxidants in cancer patients [7, 8].

*N*-acetylcysteine (NAC) and glutathione (GSH) are thiol group-containing water-soluble antioxidants. Previous research revealed the oncogenic roles of NAC or GSH in different cancer types. Exogenous antioxidants exert their functions by directly neutralizing ROS through their thiol (-SH) functional group or indirectly generating precursor molecules for de novo synthesis of intracellular antioxidants [9]. It has been reported that GSH is essential for tumor initiation in breast cancer [10]. NAC exacerbated tumor growth in lung cancer and melanoma [11, 12]. However, the effect of exogenous supplementation of NAC or GSH on liver cancer is unknown.

Hepatocellular carcinoma (HCC) is the third leading cause of cancer related-death worldwide [13]. In this study, we aimed to systematically examine the effects of antioxidant supplements in HCC using comprehensive sets of in vitro and in vivo models. Here, we show that NAC and GSH promoted HCC formation, enhanced tumor growth, upregulated stemness gene expression, and counteracted the therapeutic effects of Sorafenib. In addition, NAC and GSH regulated common biological pathways in promoting HCC formation and growth. Furthermore, sulfasalazine (SSA) and buthionine sulphoximine (BSO), which are inhibitors targeting NAC and GSH metabolism respectively, suppressed the tumor promoting effects induced by NAC and GSH in vitro and in vivo, and accompanied with significant increase in ROS level. Our study provides scientific insights regarding the use of exogenous antioxidant supplements in cancer patients.

## Materials and methods

### Chemicals

NAC, GSH and diethylnitrosamine (DEN) were obtained from Sigma-Aldrich (St. Louis, MO, USA).

### Cell lines

Human HCC cells, HepG2, Hep3B, PLC/PRF/5 (PLC) and mouse hepatoma cell line Hepa1-6 were obtained from American Type Culture Collection. Human HCC cell line MHCC-97L was a gift from Dr ZY Tang (Fudan University, Shanghai, China) and the STR authentication was conducted. HepG2, Hep3B and PLC/PRF/5 were cultured in MEM containing 10% fetal bovine serum (FBS), while the others were cultured in DMEM supplemented with 10% FBS.

### NAC and GSH and dose equivalents

High doses of NAC (120 mg/kg/day) and GSH (100 mg/kg/day) given to the animals were equivalent to 600 mg/tablet/day (10 mg/kg/day) and 500 mg/tablet/day (8.3 mg/kg/day) for a 60-kg human adult, respectively, as calculated from FDA-recommended conversion factor (12.3 for mouse) from human equivalent dose.

### Animal models

To evaluate tumor formation ability, limiting dilution assay was conducted by injecting 2 × 10^3^, 2 × 10^4^ and 2 × 10^5^ MHCC-97L cells subcutaneously into the flanks of male BALB/cAnN-nu nude mice. Vehicle control (distilled water), NAC or GSH (60 and 120 mg/kg/day for NAC, 50 and 100 mg/kg/day for GSH) was given to mice orally for 4 weeks. The tumor initiating capacity was analysed by the confidence intervals (CIs) for 1/(stem cell frequency) using extreme limiting dilution analysis. For the DEN-induced mouse model, DEN at 25 mg/kg was injected into 2-week-old C57BL/6 mice intraperitoneally to induce spontaneous HCC formation. Vehicle control (distilled water), NAC or GSH of low and high dose were given to mice orally for 35 weeks. For hydrodynamic tail-vein injection (HDTVi)-induced HCC model, sterile plasmid mix with a total volume corresponding 10% of body weight was injected into lateral tail vein of 8-week old male C57BL/6 mice in 6–8 s. A total of 30 µg of CRISPR-Cas9 vector system carrying sgRNA targeting Trp53 and transposon system carrying c-Myc vector were injected into lateral tail vein of 8–10-week old male C57BL/6 mice, as described [14]. Tumor incidence and tumor mass were recorded. Tumors were dissected and dissociated for ROS assay by flow cytometry.

Orthotopic liver injection model was employed to investigate the tumor growth, progression and stemness gene expression, Briefly, 1 × 10^6^ luciferase-labelled MHCC-97L cells were injected into the left lobes of livers of nude mice, whereas 3 × 10^6^ luciferase-labelled mouse hepatoma Hepa1-6 cells were injected into left lobes of livers of C57BL/6 mice. 2 weeks later for nude mice and 4 days later for C57BL/6 mice, all the mice were administrated with vehicle control, low and high dose of NAC or GSH for another 4 weeks for nude mice and 10 days for C57BL/6 mice, respectively.

For orthotopic liver injection HCC model, vehicle control (distilled water), Sorafenib at 10 mg/kg alone or combined with low and high dose of NAC and GSH were given to nude mice with orthotopic liver injection of luciferase-labelled 97L cells for 4 weeks. Xenogen IVIS 100 Imaging System was utilized to visualize the liver tumor size and lung metastasis. Tumors were dissected and dissociated for ROS assay by flow cytometry. Each experimental group had at least 6 mice. The body weights of mice were monitored weekly and in vivo imaging (IVIS Spectrum In Vivo Imaging System, PerkinElmer, Waltham, MA) was performed every two weeks for monitoring of the tumor size.

### Colony formation assay

Briefly, 1000 HCC cells were seeded for 24 h. Vehicle control (distilled water), NAC (1 mM) or GSH (100 µM) was added to cells for 7 days. The colonies were visualized by 0.5% of crystal violet and the numbers of colonies were counted.

### Sphere formation assay

1000 HCC cells were suspended in 0.25% methylcellulose in serum-free DMEM/F12 with or without supplementary NAC and GSH in wells pre-coated with 1% poly-HEMA to form spheroids for 7–14 days. The numbers of spheres over 100 µm in diameter were counted.

### Cell proliferation assay

3000 HCC cells were seeded in 96-well plates and treated with vehicle control (distilled water), NAC (1 mM) or GSH (100 µM). After cell fixation by DAPI, cell numbers were counted by ImageXpress Pico Automated Cell Imaging System (Molecular Devices, San Jose, California, USA) from day 1 to day 4.

### Transwell migration and invasion assays

1 × 10^5^ HCC cells were seeded in upper chamber suspended in serum-free medium for migration assay, whereas 20% of Matrigel-coated upper chamber was used for invasion assay. The lower chamber contained 500 μl DMEM medium with 10% FBS. Vehicle control (distilled water), NAC (1 mM) or GSH (100 µM) was added to upper chamber. After 18 h, the cells in upper chamber were fixed in methanol and underwent crystal violet staining. The cells having through the upper chamber were counted by software Image J (National Institutes of Health, Bethesda, USA).

### Flow cytometry for ROS and apoptosis assays

HCC cells were seeded and treated with vehicle control, sorafenib, NAC/GSH combine with Sorafenib for 48 h. For ROS assay, cells were trypsinized and stained with 2 μM chloromethyl-2′, 7′-dichlorodihydrofluorescein diacetate (CM-H2DCFDA) (Life Technologies, Carlsbad, CA, USA) and analyzed by BD FACSCanto II Analyzer (BD Science, New Jersey, USA). For apoptosis assay, cells were suspended in Annexin V binding buffer (BD Science, New Jersey, USA) containing Annexin V-FITC (MBL International, Woburn, MA, USA) and propidium iodide (BD Science) for 15 min in dark. The stained cells were measured by BD FACSCanto II Analyzer. For cell cycle analysis, cells were synchronized by Nocodazole (200 ng/mL) for 24 h and then released for another 24 h following Sorafenib treatment alone or in combination with NAC and GSH. Cells were harvested and stained by propidium iodide and subjected for flow cytometry.

### Histology

Mouse liver and lung tissues were harvested and sectioned for formalin fixation and paraffin embedding. Slides were stained with haematoxylin and eosin for histological analysis.

### Western blot analysis

Cells were lysed in sodium dodecyl sulfate and equal amounts of protein samples were separated in SDS–polyacrylamide gel electrophoresis. Membranes were incubated with primary antibody NFE2L2 (Ab62352, Abcam), GCLC (Ab 53179, Abcam), TMBIM1 (ab121358, Abcam), CLK2 (AP7530a, Abgent, San Diego, CA, USA) and α-tubulin (T9026, Sigma‐Aldrich, St. Louis, MO, USA) at 4 °C overnight. The expression of markers was detected by ECL/ECL Prime Western Blotting Detection Reagents (GE Healthcare, Piscataway, NJ, USA) following the manufacturer's instructions.

### Quantitative real-time PCR (qRT-PCR)

qRT-PCR amplification was performed using SYBR Green qPCR Master Mix (Applied Biosystems, California, USA) with specific primers (Additional file 2: Table S1).

### Transcriptome sequencing

Transcriptome sequencing (RNA-seq) of vehicle control, NAC and GSH-treated MHCC-97L cells was performed by Center for PanorOmic Sciences, The University of Hong Kong, using 101 b.p. pair-end sequencing on the HiSeq 2000 platform. Data were analyzed by TopHat-Cufflinks pipeline. Pathway analysis was performed with DAVID and Ingenuity Pathways Analysis (IPA, Qiagen, Hilden, Germany) software.

### Stable knockdown of HCC cells

A lentiviral-mediated approach was used to construct stable NRF2 or TMBIM1 knockdown HCC cell lines. Human ON‐TARGETplus SMARTpool siRNA duplexes which targeting NRF2 and non-target control were purchased from Dharmacon (Lafayette, CO). pLKO.1-puro vectors contained shRNAs targeting NRF2 (shNRF2) or TMBIM1 (shTMBIM1) and a non-target control (shNTC) were stably transduced into HCC cell lines. Puromycin selection was performed to get the stable expression of shRNAs and shNTCs. Sequences of all shRNAs are listed (Additional file 2: Table S2).

### Statistics

Statistical analyses were performed using GraphPad Prism 6.0 software (GraphPad Software Inc.). One-way ANOVA with Dunnett comparison test for more than two groups or Student’s t tests were used to compare the mean values of two groups. For in vitro functional assays, data are expressed as mean ± SD. For in vivo experiments, data are expressed as mean ± SD followed by either Unpaired t-test or Mann–Whitney test. Statistical significance was defined as *P < 0.05, ** P < 0.01, and ***P < 0.001.

### Study approval

All experimental procedures on mice were approved by the Committee on the Use of Live Animals in Teaching and Research of the University of Hong Kong (CULATR 5089-19 and 5688-21) and conducted in accordance with the Animals (Control of Experiments) Ordinance of Hong Kong.

## Results

### NAC and GSH promoted HCC tumor formation and tumor growth in spontaneous HCC mouse models and hepatoma xenografts models

**Fig. 1:**
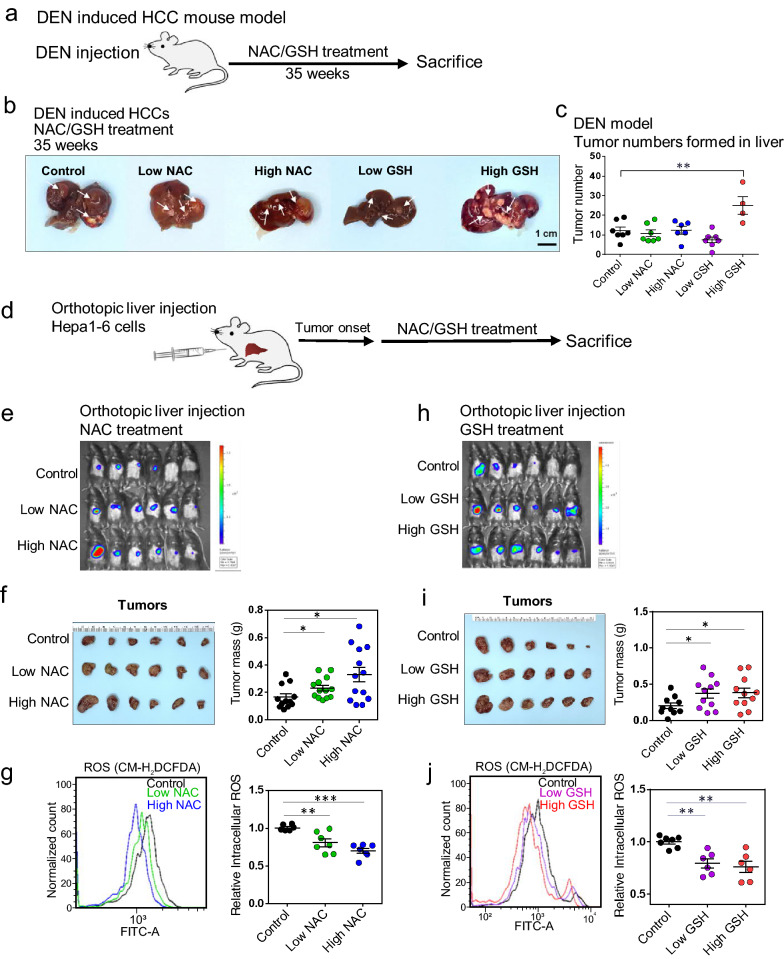
Antioxidants NAC and GSH promoted mouse HCC formation and growth.** a–b** DEN (25 mg/kg) was injected intraperitoneally into 15-day-old C57BL/6 mice, followed by feeding with NAC, GSH and vehicle control for 35 weeks. All the mice developed HCC. White arrows indicate the tumor loci formed in the livers. Scale bars: 1 cm. **c** The number of tumors formed in the NAC treatment group (left) and the GSH treatment group (right). **d** A schematic summary of the orthotopic injection model. **e** Bioluminescent images of C57BL/6 mice subjected to orthotopic liver injection of Hepa1-6 cells and treated with vehicle control, low-dose and high-dose NAC after tumor onset. **f** Liver tumors dissected from mice with NAC treatment and their tumor masses. **g** Representative flow cytometry histogram and quantification of ROS levels in vehicle control, low-dose and high-dose NAC treated tumors. **h** Bioluminescent images of C57BL/6 mice with orthotopic Hepa1-6 cell injection and treatment with vehicle control, low-dose and high-dose GSH after tumor onset. **i** Liver tumors dissected from mice having GSH treatment and their tumor masses. **j** Representative flow cytometry histogram and quantification of ROS levels in vehicle control, low-dose and high-dose GSH groups. *P < 0.05, ** P < 0.01, and ***P < 0.001 vs. control; One-way ANOVA

To investigate the effects of exogenous antioxidant treatment on tumor formation, we used the chemical carcinogen diethylnitrosamine (DEN) model to induce spontaneous HCC formation in C57BL/6 mice (Fig. 1a), followed by oral gavage of NAC and GSH with two different doses (NAC at 60 and 120 mg/kg/day and GSH at 50 and 100 mg/kg/day, respectively). The doses were biologically relevant and were estimated based on the human equivalent dose for a 60-kg adult, i.e. 600 mg/serving/day (10 mg/kg/day) for NAC and 500 mg/serving/day (8.3 mg/kg/day) for GSH divided by the 12.3, which is the recommended FDA conversion factor for mouse [15]. These doses were used in all the in vivo experiments throughout this study. After DEN injection, all mice developed multiple foci of HCC at 35 weeks (Fig. 1b). Significant body weight loss was observed in the high-dose NAC- and GSH-treated mice (Additional file 1: Fig. S1a), and the latter was accompanied by a decrease in survival rate, while NAC and low-dose GSH treatments did not affect the survival of the mice (Additional file 1: Fig. S1b). High-dose GSH treatment significantly enhanced the tumor incidence (P < 0.01), as indicated by a higher number of tumors with diameter > 1 mm formed in the livers (Fig. 1c).

To extend our investigation, we employed the hydrodynamic tail-vein injection (HDTVi) model with spontaneous HCC tumors induced by CRISPR/Cas9-mediated loss-of-function genome editing of endogenous TP53 and Sleeping Beauty (SB) transposon-driven c-Myc overexpression in C57BL/6 mice [16] (Additional file 1: Fig. S1c). Consistently, we found increased tumor incidence rate in NAC- or GSH-treated mice when compared with control group (Additional file 1: Fig. S1d), and this was accompanied with significant reduction of intra-tumoral ROS levels (Additional file 1: Fig. S1e).

Next, we questioned whether exogenous antioxidants enhanced HCC tumor growth. We used mouse tumor xenograft model by orthotopically injecting luciferase-labelled Hepa1-6, a mouse hepatoma cell line, into immunocompetent C57BL/6 mice (Fig. 1d). Significant increases in the tumor masses (Fig. 1e, f) together with a decrease in intra-tumoral ROS levels (Fig. 1g) were observed in both low- and high-dose NAC-treated groups. Similarly, with both low- and high-dose GSH treatments, along with increasing trends in the bioluminescent signals (Fig. 1h), there was a significant increase in the tumor masses (Fig. 1i). There was also a significant reduction of ROS levels (Fig. 1j). Our data suggest that NAC and GSH could promote aggressive tumor behavior by reduction of ROS levels.

### NAC and GSH exhibited pro-oncogenetic effects on human HCC cells and desensitized NRF2/GCLC related antioxidant production pathway in vitro

**Fig. 2:**
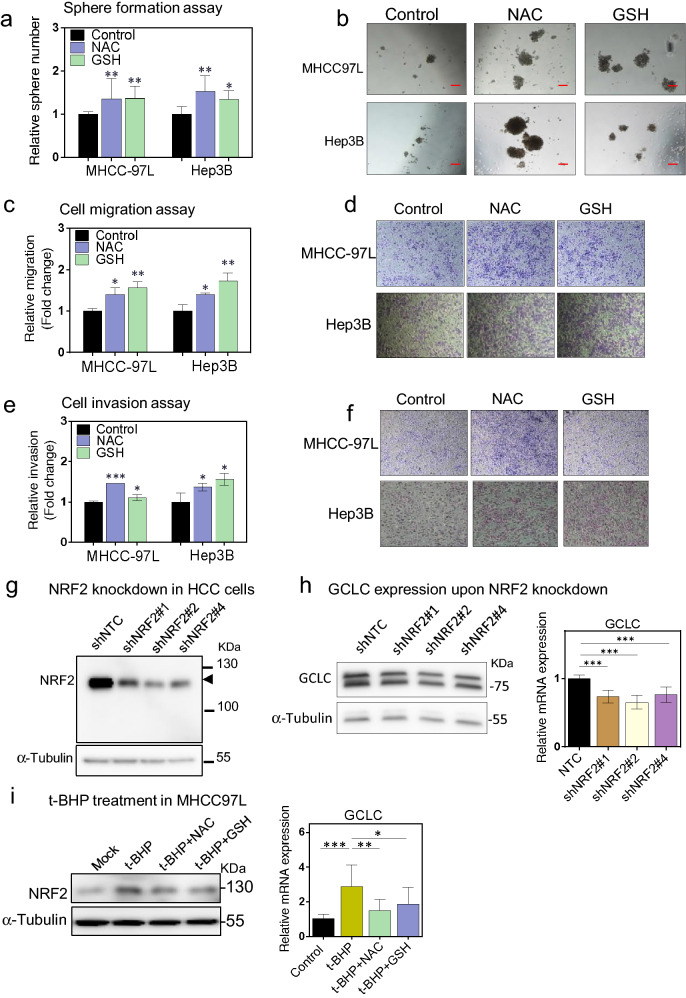
Antioxidants NAC and GSH exhibited pro-oncogenetic effects on human HCC cells and desensitized NRF2/GCLC related antioxidant production pathway in vitro. **a–b** Sphere formation assay showing the relative numbers of spheres formed of MHCC-97L and Hep3B cells in the presence of NAC and GSH as compared to the vehicle control. Results were from 3 independent experiments. **c–d** Migration rates of MHCC-97L and Hep3B cells in the presence of NAC and GSH as compared to the vehicle control. Results were from 3 independent experiments. **e–f** Invasion rates of MHCC-97L and Hep3B cells in the presence of NAC and GSH as compared to the vehicle control. Results were from 3 independent experiments. **g** NRF2 stable knockdown in MHCC-97L cells. **h** GCLC expression by qPCR and western blot upon NRF2 knockdown. **i** Expression of NRF2 (left) and GCLC (right) upon treatment with t-BHP, combination of t-BHP and NAC or GSH, and control. *P < 0.05, ** P < 0.01, and ***P < 0.001 vs. control; One-way ANOVA

Since we observed that antioxidants promoted tumorigenesis and growth in mouse spontaneous HCC and xenograft models, we wished to confirm whether these promoting effects also existed in human HCC cells both in vitro and in vivo. To systematically investigate the functional consequences of antioxidant treatment in human HCC cells, a panel of HCC cell lines, including MHCC-97L, PLC/PRF/5, Hep3B and HepG2, were challenged with NAC and GSH followed by various assays to measure their functional changes. In general, either or both of NAC and GSH promoted one or more phenotypic changes, depending on the HCC cell line tested. We found NAC or GSH promoted colony formation in MHCC-97L cells and HepG2 cells (Additional file 1: Fig. S2a), increased the cell proliferation rates (Additional file 1: Fig. S2b) and self-renewal abilities by sphere formation assay (Fig. 2a, b; Additional file 1: Fig. S2c, d) in all four cell lines, and migratory (Fig. 2c–d) and invasive abilities (Fig. 2e, f) in MHCC-97L or Hep3B cells. To further examine the pro-oncogenic effects of antioxidants in human HCC, we confined our subsequent experiments using MHCC-97L and Hep3B cells.

As NAC and GSH are thiol group (-SH)-containing antioxidants, apart from directly neutralizing ROS, they also provide precursors for endogenous GSH synthesis including cysteine, glutamine and glycine under the regulation of NRF2 (Nuclear Factor, Erythroid 2 like 2), the master gene regulator responding to cellular oxidative stress, and GCLC (Glutamate-cysteine ligase catalytic subunit), and the first rate-limited enzyme in cellular GSH biosynthesis [17, 18]. We found that the expression of Solute Carrier Family 7 Member 11 (SLC7A11) was significantly increased upon NAC and GSH treatment in HCC cells (Additional file 1: Fig. S2e). SLC7A11 is a subunit of cystine/glutamate antiporter xCT and responsible for cystine import. GSH treatment also significantly enhanced the GCLC expression (Additional file 1: Fig. S2e). Stable knockdown of NRF2 in MHCC-97L cells (Fig. 2g) reduced GCLC expression (Fig. 2h). Using tert-Butyl hydroperoxide (t-BHP), a ROS-inducer, NRF2 was activated and stabilized, and the GCLC expression was significantly upregulated correspondingly (Fig. 2i). However, exogenous supplementation of NAC or GSH reduced the expression of NRF2 and GCLC (Fig. 2i), suggesting the NRF2/GCLC-related antioxidant production pathway might be desensitized.

### NAC and GSH enhanced tumor growth and progression of human HCC cells in vivo, with accompanying attenuation of intracellular ROS levels

**Fig. 3:**
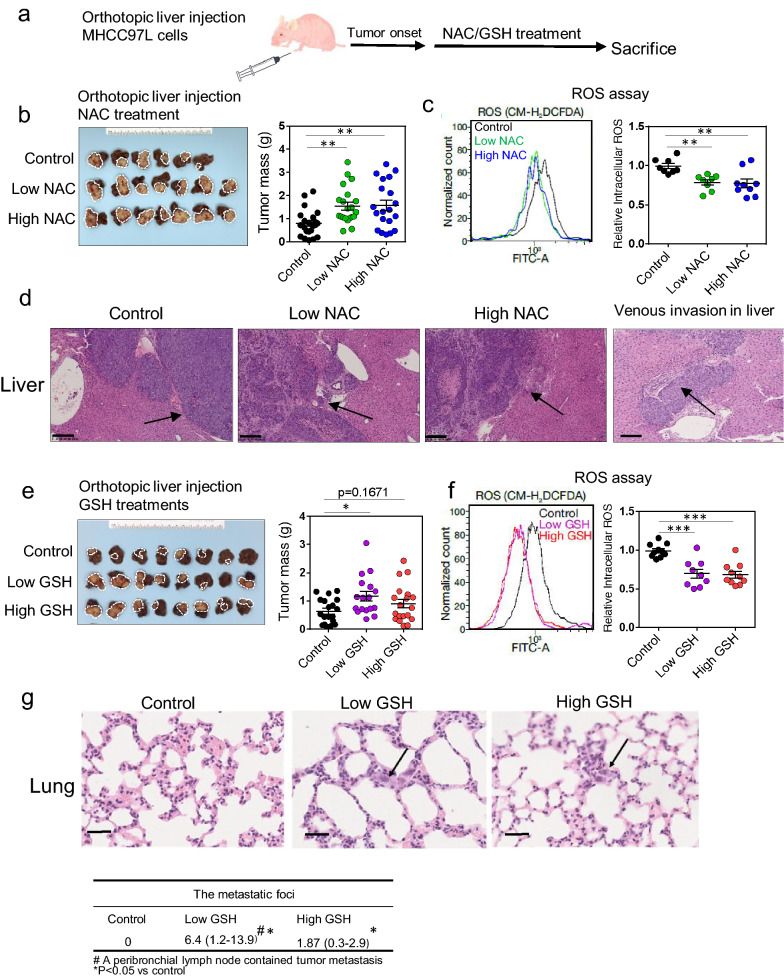
Antioxidants NAC and GSH promoted growth and progression of human HCC cells in vivo.** a** MHCC-97L-derived orthotopic HCC xenograft tumor model. **b** MHCC-97L-derived tumors and their tumor masses. **c** Representative flow cytometry histogram and quantification of the intracellular ROS levels in control, low-dose and high-dose NAC treated tumors. **d** Representative H&E sections of livers and lungs. Arrows indicate irregular tumor growth fronts. Venous invasion of HCC tumor cells is also shown (far right) (Scale bars: 250 μm). **e** MHCC-97L-derived orthotopic HCC xenograft tumors and their tumor masses with low- and high-dose GSH treatment after tumor onset in nude mice. **f** Representative flow cytometry histogram (left) and quantification (right) of ROS levels in control, low-dose and high-dose GSH treated tumors. **g** Representative H&E sections of livers and lungs. Arrows indicate tumor cells found in lung tissues (lower). The frequencies of metastatic foci in the lungs were represented by the numbers of tumor cells/foci per 10 high power fields (400 × magnification) under the microscope (Scale bars: 250 μm). Data are expressed as mean ± SD. *P < 0.05, ** P < 0.01, ***P < 0.001 vs. control, (B-G: one-way ANOVA followed by Dunnett comparison test)

To evaluate the impact of NAC or GSH on tumor growth of human HCC cells in vivo, MHCC-97L cells were orthotopically injected into the liver followed by oral administration of NAC and GSH with the two doses (Fig. 3a). We observed that both doses of NAC enhanced tumor growth as reflected by an increase in the tumor masses at the experimental endpoint (Fig. 3b; Additional file 1: Fig. S3a) and accompanied with a significant reduction in intra-tumoral ROS levels upon NAC treatment (Fig. 3c). Histological analysis further revealed more invasive tumor growth fronts in the NAC-treated groups when compared with the control (Fig. 3d). Foci of tumor venous invasion were also observed in two livers in the low-dose NAC group and one liver in the high-dose NAC group (Fig. 3d), suggesting NAC treatment could potentially promote aggressive tumor behavior. However, quantification of the metastatic foci in the lung histology sections revealed no significant difference in terms of the numbers of lung metastases (Additional file 1: Fig. S3b). On the other hand, low-dose and high-dose GSH either significantly enhanced or showed a trend in increasing tumor growth (Fig. 3e; Additional file 1: Fig. S3c) despite that both low and high doses of GSH markedly decreased the ROS levels in the tumors (Fig. 3f). More irregular fronts of xenograft tumor growth were observed in the tumors harvested from both low- and high-dose GSH-treated mice (Additional file 1: Fig. S3d). Also, significantly more metastatic foci were present in the lungs from both the low and high-dose GSH-treated groups when compared with the control group (P < 0.05) (Fig. 3g). These data indicate that exogenous antioxidants NAC and GSH may promote HCC tumorigenesis, growth and progression potentially by attenuating the intracellular ROS levels.

### Antioxidants NAC and GSH promoted tumor formation of human HCC cells in vivo

**Fig. 4:**
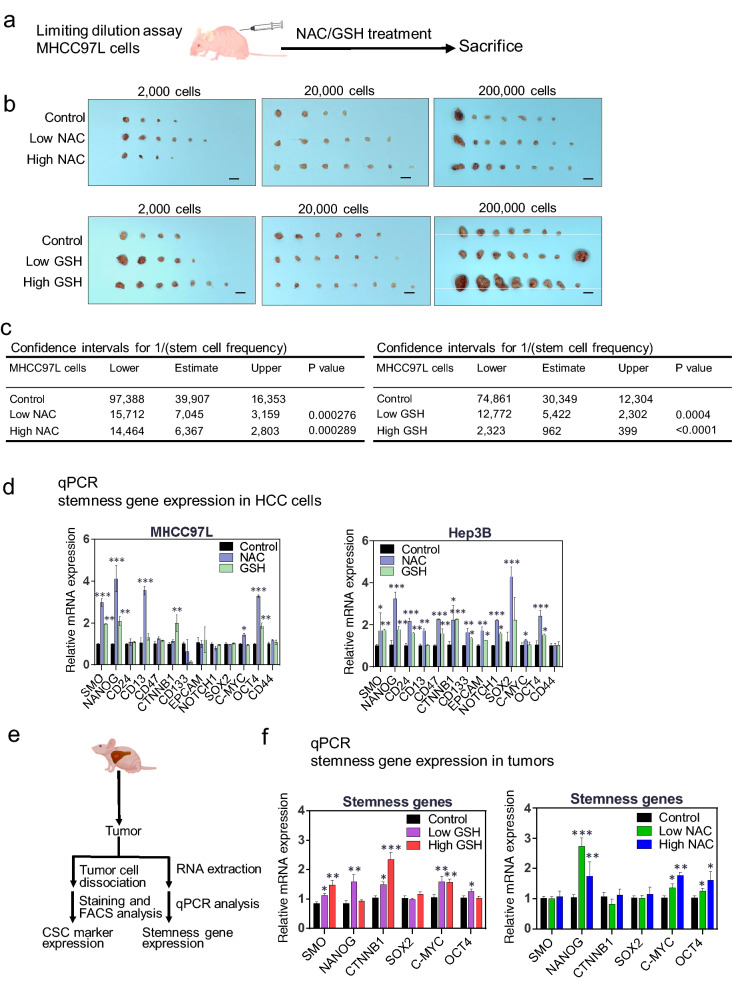
NAC and GSH enhanced tumorigenesis and promoted liver cancer stem cell markers and stemness genes. **a, b** Limiting dilution assay showing the effects of NAC and GSH on tumorigenicity in nude mice. 2 × 10^3^, 2 × 10^4^ and 2 × 10^5^ MHCC-97L cells were injected subcutaneously into nude mice. The tumor incidence rate for each group was recorded at the end of the experiments after 4 weeks. **c** Tumor initiating capacity was analyzed by the confidence intervals (CIs) with the formula of CI = 1/(stem cell frequency). **d** mRNA expression of stemness-related genes upon NAC and GSH treatment in HCC cells. **e–f** Schematic diagram illustrating the workflow of investigating the expression of CSC markers and stemness-related genes in HCC tumors harvested from mice in the orthotopic liver injection model using MHCC-97L cells. Detection of mRNA expression of stemness-related genes in tumors by qPCR with control, low- and high-dose NAC/GSH treatment. Data are expressed as mean ± SD from three independent experiments. *P < 0.05, ** P < 0.01 and ***P < 0.001 vs. control; one-way ANOVA followed by Dunnett comparison test

To investigate the effect of exogenous antioxidants on tumor formation of human HCC cells in vivo, we performed limiting dilution assay by subcutaneous injection of human HCC cells MHCC-97L into nude mice followed by NAC and GSH oral gavage, each with the two different doses (Fig. 4a). NAC treatment significantly promoted the incidence of tumor formation of MHCC-97L cells in a dose-dependent manner (Fig. 4b). In the presence of low and high doses of NAC, the estimated confidence interval (CI) for the frequency of tumorigenicity in MHCC-97L was 7045 and 6367, respectively, as compared to 39907 for the control group, indicating that NAC significantly enhanced the tumor formation frequency (P = 0.000276, P = 0.000289) (Fig. 4c; Additional file 1: Fig. S3e). Similarly, the low and high doses of GSH also significantly increased the tumor formation frequency of MHCC-97L cells (P = 0.0004 and P < 0.0001) (Fig. 4b–c; Additional file 1: Fig. S3e).

### NAC and GSH upregulated the expression of liver cancer stem cell markers and stemness genes

Since we observed that NAC and GSH treatment significantly enhanced the self-renewal ability in vitro and tumorigenicity in vivo, we then questioned whether NAC and GSH could enhance the cancer stemness properties of HCC both in vitro and in vivo. MHCC-97L and Hep3B cells were individually treated with NAC and GSH, followed by examination of the changes in the expression of liver cancer stem cell (CSC) markers and cancer stemness-related genes. Both NAC and GSH treatments significantly increased the expression of stemness-related genes SMO, NANOG and OCT-4 at mRNA levels in all treated HCC cell lines (Fig. 4d). Also, some stemness-related genes such as CD13 and C-MYC were found to be upregulated in NAC-treated HCC cells, while CD24, CD47, EPCAM, and NOTCH1 were significantly upregulated in Hep3B cells upon antioxidant treatment (Fig. 4d).

Using an in vivo model, we also examined the expression of both liver CSC markers and stemness-related genes in MHCC-97L cell-derived orthotopic liver tumors from nude mice after NAC and GSH treatment (Fig. 4e). Low-dose NAC significantly increased CD13 expression (Additional file 1: Fig. S3f). The mRNA expression of CD44 was significantly upregulated by high-dose NAC and high-dose GSH in mice (Additional file 1: Fig. S3g). Other stemness-related genes including Nanog, c-Myc and Oct4 were significantly upregulated upon low- and high-dose NAC treatment in mouse tumors (Fig. 4f). Similarly, low- and high-dose GSH treatment also promoted the expression of stemness-related genes including Nanog, c-Myc and Oct4, SMO, and β-catenin (Fig. 4f). However, there was no change in the expression of liver CSC markers by GSH (Additional file 1: Fig. S3f). Altogether, our data suggest that NAC and GSH may play a role in enhancing HCC stemness both in vitro and in vivo.

### NAC and GSH counteracted the tumor-suppressive effect of sorafenib in human HCC cells in vitro and in vivo

**Fig. 5:**
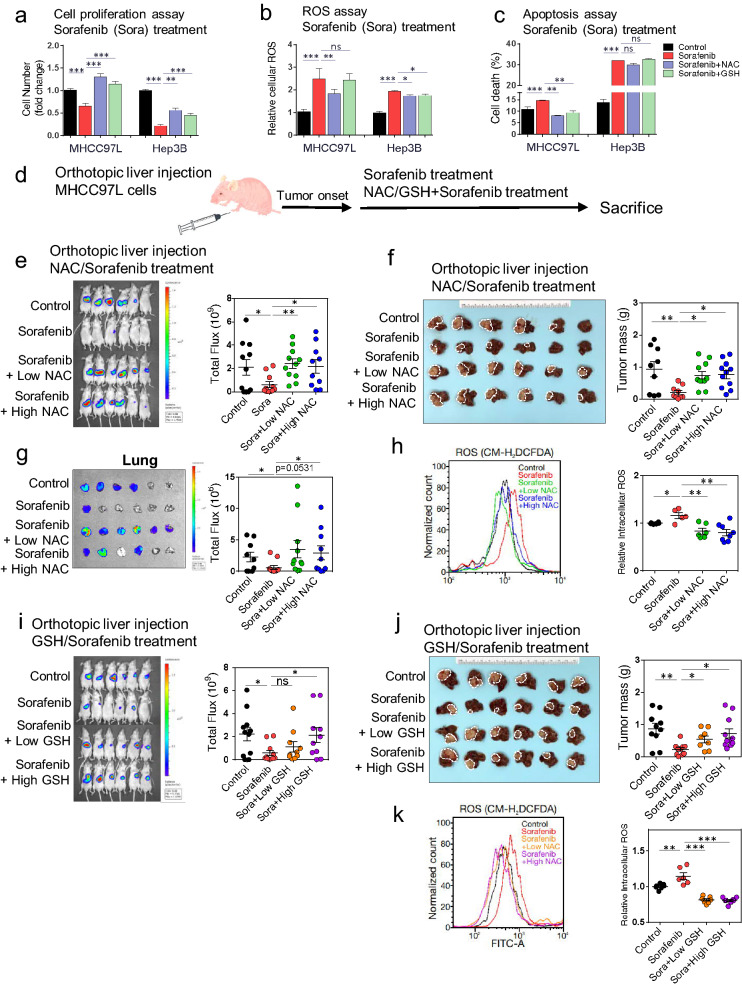
NAC and GSH counteracted the therapeutic effect of Sorafenib in human HCC both in vitro and in vivo.** a** Quantification of the relative numbers of MHCC-97L and Hep3B cells treated with Sorafenib (5 µM), combined Sorafenib and NAC (1 mM), and combined Sorafenib and GSH (100 µM). Data were normalized and compared to the control. Results represent 3 independent experiments. **b** ROS assay with CM-H2DCFDA staining. Data represent the intensities of FITC normalized with control. **c** Annexin V assay for quantification of apoptotic cell population in MHCC-97L and Hep3B cells subjected to Sorafenib (5 µM), combined Sorafenib and NAC, and combined Sorafenib and GSH treatments. The data shown are representative of three independent experiments. **d** A schematic summary of Sorafenib treatment in vivo. **e** Bioluminescent images of mice bearing orthotopic MHCC-97L cell-derived xenograft tumors with treatment of Sorafenib (10 mg/kg), and combined Sorafenib and NAC treatment at both low and high doses. **f** Liver tumors with mice treated with Sorafenib and combined Sorafenib and NAC at both low and high doses and their tumor masses. **(g)** Bioluminescent images of lung tissues and quantification of their bioluminescent intensities. **h** Representative flow cytometry histogram and quantification of intra-tumoral ROS levels in the indicated groups. **i** Bioluminescent images of mice bearing orthotopic MHCC-97L cell-derived xenograft tumors with treatment of Sorafenib (10 mg/kg), and combined Sorafenib and GSH at both low and high doses. **j** Liver tumors subjected to Sorafenib (10 mg/kg), and combined Sorafenib and GSH treatment at both low and high doses and their tumor masses. **k** Representative flow cytometry histogram and quantification of ROS levels in vehicle control, Sorafenib alone and combination groups. Data are expressed as mean ± SD. *P < 0.05, ** P < 0.01, and ***P < 0.001 vs. Control or Sorafenib; one-way ANOVA followed by Dunnett comparison test

Given that NAC and GSH may enhance the aggressiveness of HCC cells, we questioned whether exogenous antioxidants would thus lower the responsiveness of HCC cells towards Sorafenib treatment, one of the first-line molecular targeted drugs for patients with advanced HCC [19]. When compared to the stand-alone Sorafenib treatment, combined treatment of either NAC or GSH and Sorafenib abrogated the suppressive effects on cell proliferation by Sorafenib in MHCC-97L and Hep3B cells (Fig. 5a). The Sorafenib-induced increase in ROS level (Fig. 5b) and cell death (Fig. 5c; Additional file 1: Fig. S4a) were reduced upon addition of NAC and GSH in MHCC-97L and/or Hep3B cells (Fig. 5a–c, Additional file 1: Fig. S4a). Furthermore, cell cycle analysis by flow cytometry showed that the NAC and GSH treatment reduced the Sorafenib-induced G1-phase cell cycle arrest in MHCC-97L and increased the cell population at G2/M phase (Additional file 1: Fig. S4b).

We further assessed the impact of NAC and GSH on Sorafenib treatment in vivo using the orthotopic liver injection model with MHCC-97L cells in nude mice (Fig. 5d). At 10 mg/kg, Sorafenib effectively suppressed the tumor growth; interestingly, addition of NAC at both low and high doses effectively abrogated such growth suppression (Fig. 5E and Additional file 1: Fig. S4c–d), as indicated by the significant increase in tumor masses (Fig. 5f) and lung metastases (Fig. 5g; Additional file 1: Fig. S4d) when compared to the Sorafenib treatment alone. In line with our in vitro results, combined treatment groups showed comparatively lower intratumoral ROS levels than the Sorafenib-alone group (Fig. 5h). GSH treatment at both low and high doses exhibited similar antagonizing effects to Sorafenib-mediated tumor suppression (Fig. 5i, j, and Additional file 1: Fig. S4e). However, no difference in the degree of lung metastasis was observed among the stand-alone sorafenib treatment and the combined treatment groups with GSH (Additional file 1: Fig. S4f), despite the intra-tumoral ROS levels were significantly reduced by GSH at both doses (Fig. 5k).

### NAC and GSH altered common molecular pathways to affect metabolism and epigenetics

We questioned whether their underlying actions were mediated through common molecular pathways. To this end, MHCC-97L cells were individually treated with NAC and GSH, respectively, followed by whole transcriptomic sequencing (RNA-seq). By extracting differentially expressed genes ≥ 2 folds, 155 and 162 genes were identified upon NAC and GSH treatment, respectively. Eighty-six and 64 genes of them were found to be upregulated, respectively, while 69 and 88 of them were found to be downregulated, respectively (Additional file 1: Fig. S5a). We subjected the differentially expressed genes to gene set enrichment analysis (GSEA) for potential biological processes altered by the antioxidant treatments (Additional file 1: Fig. S5b). Genes that were significantly co-upregulated by NAC and GSH were enriched in negative regulation of carbohydrate metabolic process and vocalization behaviour, which are associated with organism respiratory system (Additional file 1: Fig. S5b). Besides, NAC treatment also upregulated genes in several cellular metabolic processes, including modified amino acid metabolism and S-adenosylhomocysteine metabolism (Additional file 1: Fig. S5b), which play crucial roles in donating homocysteine during methionine metabolism and further promote endogenous GSH synthesis to combat ROS [20]. On the other hand, the co-downregulated genes in NAC and GSH treatment groups were mainly associated with epigenetic alterations, including epigenetic gene expression, chromatin assembly and DNA silencing (Additional file 1: Fig. S5c).

### TMBIM1 was upregulated upon NAC and GSH treatment and knockdown TMBIM1 neutralized the oncogenetic effect caused by antioxidants in vitro

**Fig. 6:**
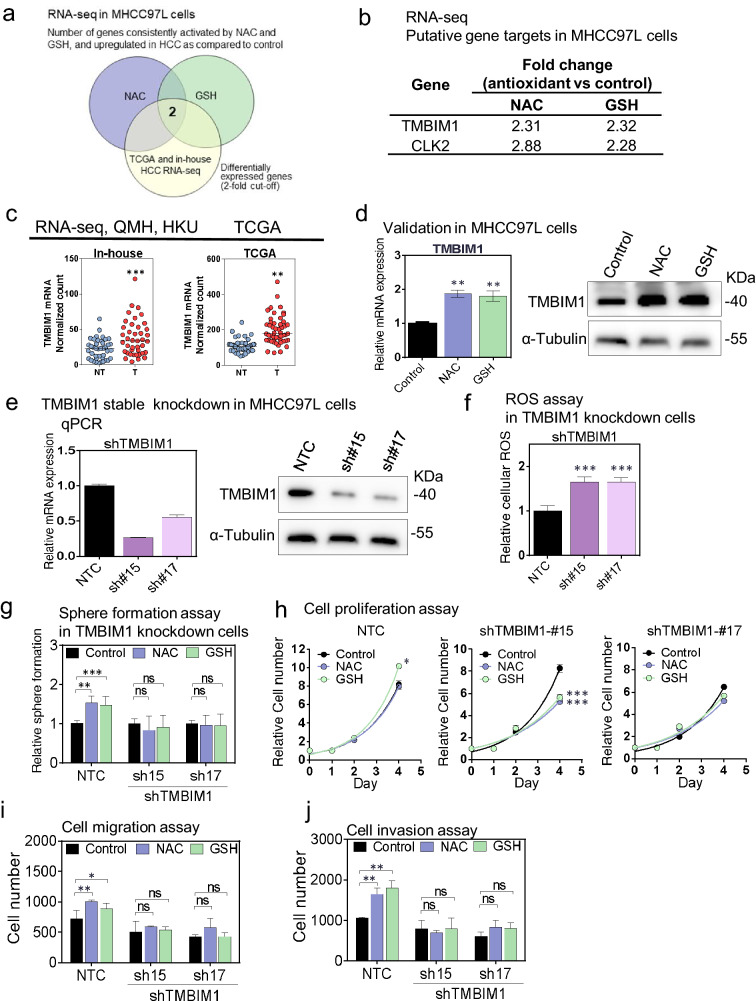
TMBIM1 was upregulated in HCC upon antioxidants treatment.** a, b** Diagrams showing number and the fold change of genes consistently activated by NAC and GSH, and upregulated in our cohort and TCGA HCC as compared to control by whole transcriptome sequencing (> 2 folds compared to control and consistent trend between NAC and GSH treatment). **c** TMBIM1 and CLK2 expression in NT (non tumor) and T (tumor) of HCC patients. **d** qPCR and western blot validation of TMBIM1 upregulation on MHCC-97L cells. **e** Stable knockdown of TMBIM1 in HCC cells. **f** ROS assay in NTC and TMBIM1 knockdown cells. **g–i** Sphere formation assay, cell proliferation assay, cell migration and invasion assay in TMBIM1 knockdown cells. Error bars indicate mean ± SD. *P < 0.05, **P < 0.01, ***P < 0.001 vs. NTC or control. one-way ANOVA followed by Dunnett comparison test

Among the deregulated genes after treatment with exogenous antioxidants, Transmembrane BAX Inhibitor Motif Containing 1 (TMBIM1) and CDC Like Kinase 2 (CLK2) mRNA were significant upregulated upon NAC and GSH treatments (Fig. 6a, b) and these 2 genes were also significantly overexpressed in both TCGA database and our in-house RNA-seq database on 41 pairs of clinical HCC patients (Fig. 6c and Additional file 1: Fig. S6a). TMBIM1 is a multi-pass membrane protein and located in cell membrane, lysosome and endosome membrane [21]. Previous research revealed TMBIM1 maintained cellular Ca^2+^ homeostasis and cell survival by inhibiting FasL-mediated apoptosis in vascular diseases [22]. Another research team has demonstrated TMBIM1 exerted anti-inflammatory protective role for chronic liver diseases including non-alcoholic fatty liver disease (NAFLD) via degradation of the TLR4 (Toll-like receptor 4) [23]. Of note, there is no comprehensive study about the effects of TMBIM1 on cancer formation and growth. Besides, the relationship between TMBIM1 and antioxidants in cancer is unknown. The oncogenetic kinase effect of CLK2 was reported previously in other cancer types such as breast cancer by regulating the mRNA splicing [24, 25], however the protein level of CLK2 was not alter upon antioxidant treatment in MHCC97L (Additional file 1: Fig. S6b). Therefore we focussed our further investigation on TMBIM1 in the present study. First, the upregulation of TMBIM1 by NAC and GSH was validated in MHCC97L cells at mRNA and protein level (Fig. 6d). Stable knockdown of TMBIM1 significantly increased the intracellular ROS level in MHCC97L cells, suggesting that overexpression of TMBIM1 could suppress the oxidative stress in HCC (Fig. 6e, f). Then we treated the TMBIM1-knockdown cells with exogenous antioxidants NAC and GSH. Upon TMBIM1 knockdown, the promoting effects of the antioxidants on sphere formation (Fig. 6g), cell proliferation (Fig. 6h), migration and invasion (Fig. 6i, j) was abolished as compared with shNTC (Additional file 1: Fig. S6c–f), respectively. Our data suggest that TMBIM1 may be a potential downstream target of exogenous antioxidant-induced HCC exacerbation.

### Inhibitors targeting GSH and NAC metabolism abrogated the pro-oncogenic effects exerted by NAC and GSH

**Fig. 7:**
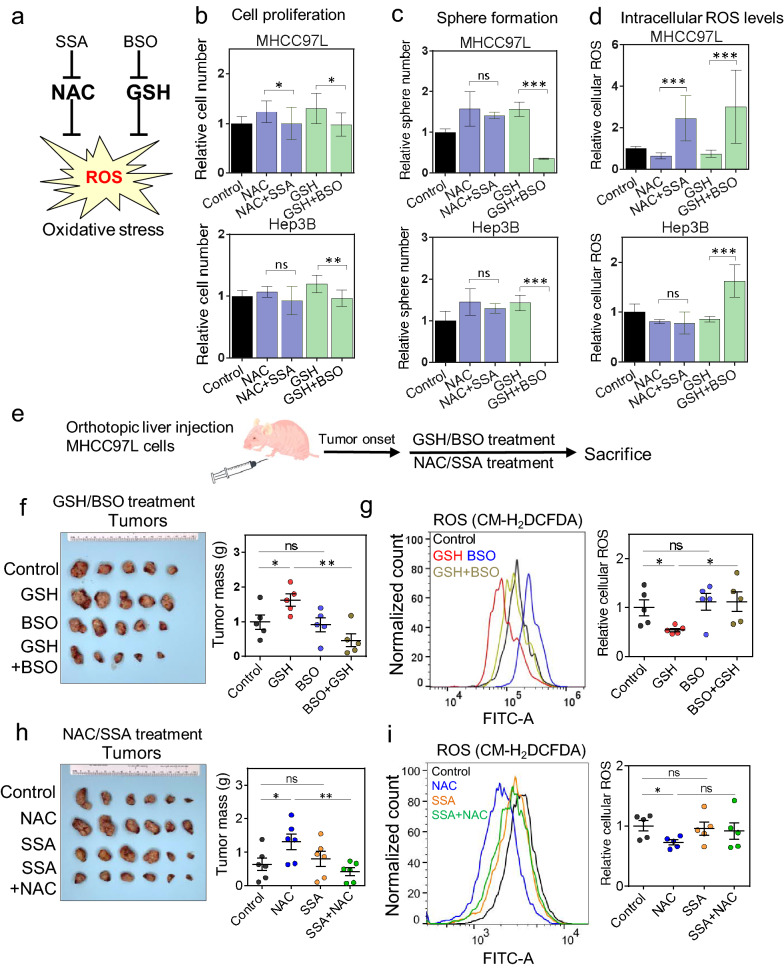
Inhibitors targeting GSH and NAC metabolism abrogated the pro-oncogenic effects exerted by NAC and GSH.** a** A schematic summary of SSA and BSO. **b** The relative cell numbers, (**c**) the relative numbers of spheres, and (**d**) relative ROS levels of MHCC-97L and Hep3B cells after treatment with NAC, GSH and the indicated inhibitors. Data were normalized and compared to the control and expressed as mean ± SD from three independent experiments. *P < 0.05, ** P < 0.01, and ***P < 0.001; Unpaired t-test. **e** A schematic summary of BSO treatment in vivo. **f** MHCC-97L-derived orthotopic HCC xenograft tumors and their tumor masses after GSH, BSO and BSO + GSH treatment after tumor onset in nude mice. **g** Representative flow cytometry histogram and quantification of the intracellular ROS levels in control, GSH, BSO, BSO + GSH treated tumors. **h** MHCC-97L-derived orthotopic HCC xenograft tumors and their tumor masses after NAC, SSA and SSA + NAC treatment after tumor onset in nude mice. **i** Representative Flow cytometry histogram and quantification of the intracellular ROS levels in control, NAC, SSA, SSA + NAC treated tumors. Error bars indicate mean ± SD. *P < 0.05, **P < 0.01, ***P < 0.001 vs. NTC or control. one-way ANOVA followed by Dunnett comparison test

We questioned whether targeting GSH synthesis or cystine transportation could neutralize or reverse the pro-oncogenic effects driven by these antioxidants. To deplete cellular GSH, Buthionine-sulfoximine (BSO), a GSH synthase inhibitor, was used (Fig. 7a; Additional file 1: Fig. S7a), while Sulfasalazine (SSA), an inhibitor of SLC7A11, which is a subunit of cystine-glutamate antiporter xCT, was used to abolish cystine absorption and importation (Fig. 7a; Additional file 1: Fig. S7b).

Individual or combined SSA and BSO were able to abolish the enhanced cell proliferation with either NAC or GSH treatment in MHCC-97L and Hep3B cells (Fig. 7b and Additional file 1: Fig. S7c). Besides, BSO but not SSA inhibited the sphere formation ability in these HCC cell lines (Additional file 1: Fig. S7d). Interestingly, individual or combined SSA and BSO treatment significantly inhibited sphere forming ability in the presence of GSH (Fig. 7c). As expected, the addition of BSO, SSA or both increased the intracellular ROS levels in HCC cells (Additional file 1: Fig. S7e). Of note, BSO and SSA abrogated the reduction of ROS by GSH and NAC in MHCC-97L cells (Fig. 7d), and BSO reversed the ROS-reducing effect of GSH in Hep3B cells (Fig. 7d).

**Fig. 8:**
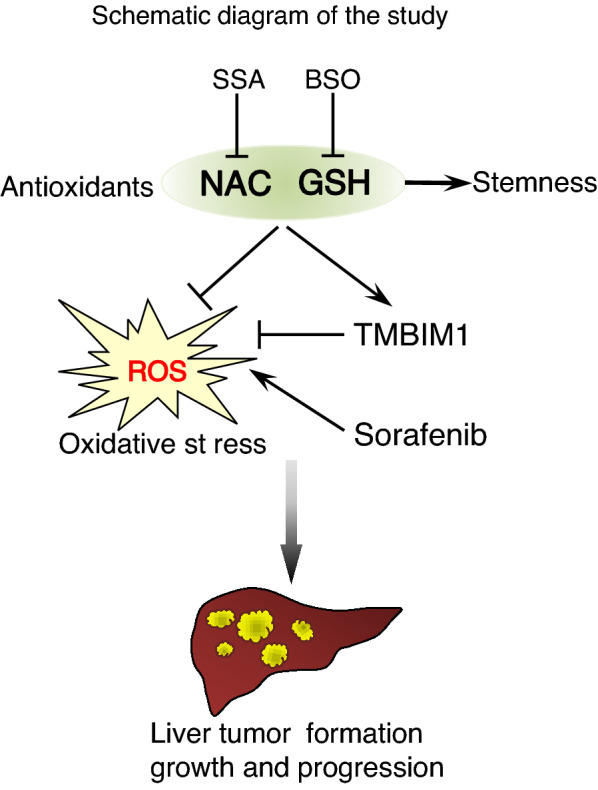
A schematic summary of the findings of antioxidant-induced HCC exacerbation in this study

Using an in vivo mouse model (Fig. 7e), BSO was able to inhibit the tumor growth enhanced by GSH while BSO on its own did not alter the tumor growth (Fig. 7f and Additional file 1: Fig. S7f). Intra-tumoral ROS level was significantly decreased upon GSH administration, while BSO, whether alone or combined with GSH, abolished this ROS-reducing effect (Fig. 7g). There were fewer lung metastases in BSO and BSO + GSH group (Additional file 1: Fig. S7g). Similarly, in vivo treatment with NAC + SSA significantly abrogated the tumor promoting effect of NAC in mice while SSA itself did not have inhibitory effects in tumor growth (Fig. 7h and Additional file 1: Fig. S7h). There was a trend of increased intra-tumoral ROS level of NAC + SSA combo group when compared with NAC (Fig. 7i). Lung metastasis signals were reduced by addition of SSA to NAC, while the incidence of lung metastasis was not altered (Additional file 1: Fig. S7i). In summary, our data implicate that NAC and GSH as exogenous antioxidants promote HCC formation, enhance tumor growth, and counteract the therapeutic effect of Sorafenib both in vitro and in vivo by reducing the intracellular ROS levels and desensitizing NRF2/GCLC-related antioxidant production pathways. TMBIM1 may be a potential downstream target of exogenous antioxidant in HCC exacerbation (Fig. 8).

## Discussion

We previously demonstrated that intracellular ROS levels are precisely modulated by HCC cells through multiple mechanisms. For instance, upregulation of transketolase (TKT), a critical enzyme in the pentose phosphate pathway, can increase the production of endogenous NADPH which serves as an electron donor to reduce the ROS [26]. Besides, HCC cells could reduce the mitochondrial activity under hypoxia, by inducing the expression of NDUFA4L2 to limit oxygen consumption and the subsequent building of intracellular oxidative stress [27]. Also, thioredoxin reductase 1 (TXNRD1), a key enzyme in the thioredoxin system in actively catalyzing the generation of cellular antioxidants to combat against ROS, was overexpressed in HCC [14]. In addition, Facilitates Chromatin Transcription (FACT) complex could mediate the epigenetic activation and stabilization of NRF2, a master transcription regulator of oxidative stress response, in supporting HCC progression [28]. As antioxidants may play a supportive role in cancer formation and progression, the intake of antioxidants as casual diets or health care supplements should be carefully re-evaluated.

In the current study, we found that NAC and GSH exerted a tumor-promoting effect in vitro and in vivo. Using multiple mouse models of chemically-induced HCC, spontaneous HCC (by genome-editing) and xenograft models, both antioxidants promoted HCC tumor formation and growth, and this was accompanied with significant reduction of intracellular ROS levels. Moreover, NAC and GSH promoted cancer stemness as reflected by upregulation of liver CSC markers and stemness-related genes. However, high doses of NAC and GSH were well tolerated and non-toxic in non-tumor bearing nude mice and C57BL/6 immunocompetent mice as reflected by the steady bodyweight and liver/body ratio throughout the experiments (Additional file 1: Fig. S8a–d). Of note, exogenous supplementation of NAC or GSH reduced the expression of NRF2 and GCLC, suggesting the NRF2/GCLC-related antioxidant production pathway might be desensitized in MHCC-97L cells which was STR authenticated (Additional file 1: Fig. S9).

Recently, some studies have provided evidence that activation of GSH production or thioredoxin system in cancer cells might exert counteractive effects on cancer treatments including chemotherapy [29, 30], while targeting the antioxidant enzyme such as catalase could inhibit CSCs in breast cancer and improve therapeutic efficacy [31]. For advanced HCC, Sorafenib is one of the first-line drugs [32]. Of significance, in this study, both NAC and GSH could abrogate the growth-suppressive effects exerted by Sorafenib in vitro in tumor xenografts. Our observations strongly suggest that the antioxidants could produce a counteractive effect on Sorafenib treatment. It would be important to evaluate whether patients with HCC should avoid unnecessary antioxidant supplements intake during Sorafenib treatment. Further investigations are warranted.

Using transcriptome sequencing followed by functional assays, we identified that TMBIM1 was a downstream target induced by NAC and GSH. Stable knockdown of TMBIM1 significantly increased the intracellular ROS in HCC cells. The promoting effect caused by antioxidants, including enhanced sphere formation, cell proliferation, migration and invasion, was neutralized or reversed upon TMBIM1 knockdown in HCC cells. Our data suggest that TMBIM1 is a potential downstream target of exogenous antioxidants-induced tumorigenesis in HCC. Reports on TMBIM1 are scanty. It has been reported that TMBIM1 forms a complex with apoptosis receptor Fas/CD95/Apo1 at Golgi apparatus and inhibits cell death [21, 33]. Another study on cardiomyopathy revealed that TMBIM1 deficiency exacerbated the inflammation and oxidative stress induced by high fat diet by reducing Nrf2 and Ho-1 while increasing Keap-1 expression in mice [34]. To our knowledge, there is no comprehensive study about the effects of TMBIM1 on tumorigenesis and growth. In addition, the relationship between TMBIM1 and antioxidants in cancer is unknown. Further investigation on the role of TMBIM1 in cancer would be warranted.

Cellular import of cysteine is the rate-limiting step for de novo GSH synthesis [35]. Targeting cysteine transportation by SSA, glutathione synthases by BSO, and thioredoxin production by Auranofin showed a significant anti-tumor response in breast cancer [10, 36]. In liver cancer, SSA was reported to inhibit CD133-positive HCC cells and sensitize HCC cells towards chemotherapy [37]. Furthermore, the direct ablation of GSH synthesis by BSO has also been demonstrated to exert therapeutic effects in various types of cancer cells [38, 39]. In this study, we demonstrated that depletion of GSH and cysteine by BSO and SSA in HCC cells could partially suppress the aggressive phenotypes induced by GSH and NAC. Furthermore, the ROS scavenging effects by NAC and GSH could be effectively abolished by BSO alone or a combination of BSO and SSA. We therefore attempted to further explore some potential HCC therapy and performed cell proliferation assay (Additional file 1: Fig. S10a) in HCC cell lines with treatments of control, sorafenib, sorafenib + SSA, sorafenib + BSO, and sorafenib + SSA + BSO. Interestingly, only sorafenib + SSA + BSO significantly inhibited cell proliferation when compared with sorafenib single treatment; the finding is consistent with that of a previous breast cancer study that SSA and BSO showed synergistic inhibitory effect in tumor cells [40]. Our data strongly suggest that targeting the cellular machinery responsible for generating antioxidants could distort the redox balance in HCC cells, leading to the accumulation of ROS and retarding HCC exacerbation.

## Conclusions

Our study showed that exogenous antioxidants NAC and GSH, by reducing the intracellular ROS levels and inducing TMBIM expression, promoted HCC formation and tumor growth, and counteracted the therapeutic effect of Sorafenib both in vitro and in vivo. Inhibitors that target NAC and GSH metabolism reversed these pro-oncogenic effects. Our findings have thus provided scientific insights regarding the implication of antioxidant supplements intake in HCC patients.

## Supplementary Information

**Additional file 1: Fig S1**. **(a)** The body weight changes upon NAC and GSH treatment in DEN-induced HCC mouse model. Data are expressed as mean ± SD from three independent experiments. **(b)** Survival rates of mice in NAC- and GSH-treated group when compared with control. **(c)** Hydrodynamic tail vein injection (HDTVi) (p53 KO/c-Myc) induced spontaneously tumorigenesis. **(d)** Tumor incidence of HDTVi (p53 KO/c-Myc) tumors in the presence of NAC treatment (upper panel) or GSH treatment (lower panel). ‘n’ represents the number of mice in each group. The tumor incidence was defined by the numbers of liver tumors > 1 mm in diameter. **(e)** Representative flow cytometry histogram (upper) and quantification (lower) of ROS levels in control, low-dose and high-dose NAC groups (left panel), and low- and high-dose GSH groups (right panel). Data are expressed as mean ± SD from three independent experiments. *P < 0.05, ** P < 0.01, and ***P < 0.001 vs. control; one-way ANOVA followed by Dunnett comparison test. **Figure S2**. **(a)** Colony formation assay in multiple HCC cell lines (MHCC7L, PLC/PRF/5, Hep3B and HepG2) with addition of NAC (1 mM), GSH (100 μM) and vehicle control. **(b)** Cell proliferation assay in MHCC-97L, HepG2, Hep3B and PLC/PRF/5 cells with treatment of NAC and GSH and in vehicle controls. **(c)-(d)** Sphere formation assay in HepG2 and PLC/PRF/5 cells with addition of NAC, GSH and control. **(e)** mRNA expression of SLC7A11 and GCLC by qPCR upon NAC and GSH treatment in MHCC97L cells. Data are expressed as mean ± SD from three independent experiments. *P < 0.05, **P < 0.01, ***P < 0.001 vs. NTC or control. one-way ANOVA followed by Dunnett comparison test. **Figure S3**. **(a)** Bioluminescent images of mice with orthotopic MHCC-97L cell injection and treated with control, low (60 mg/kg) and high dose (120 mg/kg) of NAC after tumor onset. **(b)** Representative H&E sections of lungs. Arrows indicate tumor cells found in lung tissues (lower). The frequencies of metastatic foci in the lungs were represented by the number of tumor cells/foci per 10 high power fields (× 400 magnification). **(c)** Bioluminescent images of mice with orthotopic MHCC-97L cell injection and treated with control, low (50 mg/kg) and high dose (100 mg/kg) of GSH after tumor onset. **(d)** Representative pictures of H&E sections of liver. Arrows indicate irregular growth fronts. **(e)** The tumor incidence rate for each group was recorded at the end of the experiments after 4 weeks. Tumor initiating capacity was analyzed by the confidence intervals (CIs) with the formula of CI = 1/(stem cell frequency). **(f)** Expression of liver CSC markers by flow cytometry upon control, low- and high-dose NAC/GSH treatment in nude mice. **(g)** Expression of CD44 mRNA by qPCR in the tumors of the orthotopic liver injection model upon low- and high-dose NAC/GSH treatment. Data are expressed as mean ± SD from three independent experiments. *P < 0.05, **P < 0.01, ***P < 0.001 vs. NTC or control. one-way ANOVA followed by Dunnett comparison test. **Fig. S4**. **(a)** Apoptosis assay by flow cytometry. **(b)** Cell cycle analysis by flow cytometry. **(c)** Bioluminescent images of liver tissues in mice treated with control, Sorafenib (10 mg/kg) alone, and combination of Sorafenib and low-dose (60 mg/kg) or high-dose (120 mg/kg) NAC. **(d)** Representative histology of livers. Arrows indicate irregular growth fronts of tumors (upper panel) (scale bars, 250 μm). The frequencies of metastatic foci in the lungs were represented by the numbers of tumor cells/foci per 10 high power fields (× 400 magnification) under the microscope. **(e)** Bioluminescent images of liver tissues in mice treated with control, Sorafenib (10 mg/kg) alone, and combination of Sorafenib and low-dose (50 mg/kg) or high-dose (100 mg/kg) GSH. **(f)** Bioluminescent images of lung tissues (left) and quantification of bioluminescent intensities of lung tissues (right). **(g)** Representative histology of livers. Arrows indicate irregular growth fronts of tumors (upper panel) (scale bars, 250 μm). The frequencies of metastatic foci in the lungs were represented by the numbers of tumor cells/foci per 10 high power fields (× 400 magnification) under the microscope. Data is expressed as mean ± SD from three independent experiments. *P < 0.05, **P < 0.01, ***P < 0.001 vs. NTC or control. one-way ANOVA followed by Dunnett comparison test. **Fig S5**. **(a)** Diagrams showing the total numbers of differentially expressed genes detected upon NAC and GSH treatment in MHCC-97L cells by whole transcriptome sequencing (> 2 folds compared to control and consistent trend between NAC and GSH treatment). **(b)-(c)** Gene set enrichment analyses (GSEA) identified the up-regulated and down-regulated pathways enriched in NAC and GSH treated MHCC-97L cells. **Fig. S6**. **(a)** CLK2 expression in clinic HCC patients**. (b)** CLK2 expression upon NAC and GSH treatments in MHCC-97L cells. **(c)-(f)** Sphere formation, cell proliferation, migration and invasion assays in NTC and TMBIM1 knockdown cells. Data is expressed as mean ± SD from three independent experiments. *P < 0.05, ** P < 0.01 and ***P < 0.001 vs. control or NTC; one-way ANOVA followed by Dunnett comparison test. **Fig. S7**. **(a)-(b)** BSO or SSA suppressed glutathione and cysteine levels in HCC cells. **(c)** Cell proliferation assay, **(d)** Sphere formation assay, and **(e)** ROS assay in MHCC-97L and Hep3B cells with control and treatment with SSA, BSO, and combination of SSA and BSO in MHCC-97L and Hep3B cells. **(f)** Bioluminescent images of mice treated with control, GSH, BSO and combination of GSH and BSO and the quantification of the bioluminescent signals. **(g)** Quantification of lung bioluminescent intensities treated with control, GSH, BSO and combination of GSH and BSO. **(h)** Bioluminescent images of mice treated with control, NAC, SSA and combination of NAC and SSA. **(i)** Lung bioluminescent intensities treated with control, NAC, SSA and combination of NAC and SSA. A-E: Data are expressed as mean ± SD from three independent experiments. *P < 0.05, ** P < 0.01 and ***P < 0.001 vs. control; one-way ANOVA followed by Dunnett comparison test. **Fig. S8. (a)** Nude mice were fed with high-dose NAC and GSH and vehicle control for 6 weeks. **(b)** The body weights, liver weights and liver/body ratios showed no significant different in the 3 groups. **(c)** C57BL/6 mice were fed with NAC and GSH, both at high doses, and vehicle control for 35 weeks. **(d)** The body weights, liver weights and liver/body ratios showed no significant different in the 3 groups, except the body weight in the high-dose NAC group. Data is expressed as mean ± SD. *P < 0.05, ** P < 0.01, and ***P < 0.001 versus control, one-way ANOVA followed by Dunnett comparison test. **Fig. S9.** STR authentication report of MHCC-97L cells. **Fig. S10. (a)** Cell proliferation assay in HCC cell lines with treatments of sorafenib, sorafenib + SSA, sorafenib + BSO, and sorafenib + SSA + BSO. Data are expressed as mean ± SD from three independent experiments. *P < 0.05, ** P < 0.01 and ***P < 0.001 vs. control or Sorafenib; one-way ANOVA followed by Dunnett comparison test.

**Additional file 2**:** Table S1** Primer sequences used for qRT-PCR analysis. **Table S2.** Stable sh-knockdown sequences used in this study.

## Data Availability

All data presented or analysed in present study are included in this manuscript.

## Abbreviations

NAC: *N*-Acetylcysteine
GSH: Glutathione
DEN: Diethylnitrosamine
Trp53KO/C-MycOE: P53 knock out and c-Myc overexpression
NRF2: Nuclear factor-erythroid factor 2-related factor 2
t-BHP: Tert-butyl hydroperoxide
GCLC: Glutamate-cysteine ligase catalytic subunit
SLC7A11: Solute carrier family 7 member 11
TMBIM1: Transmembrane BAX inhibitor motif containing 1
CLK2: CDC like kinase 2
NTC: Non-target control
BSO: Buthionine sulphoximine
SSA: Sulfasalazine
