# Supplementary material for: Antioxidant supplements promote tumor formation and growth and confer drug resistance in hepatocellular carcinoma by reducing intracellular ROS and induction of TMBIM1
Source: Cell Biosci. 2021 Dec 19;11:217. doi: 10.1186/s13578-021-00731-0 (PMC8684635; doi:10.1186/s13578-021-00731-0)

Supplementary Figure 1

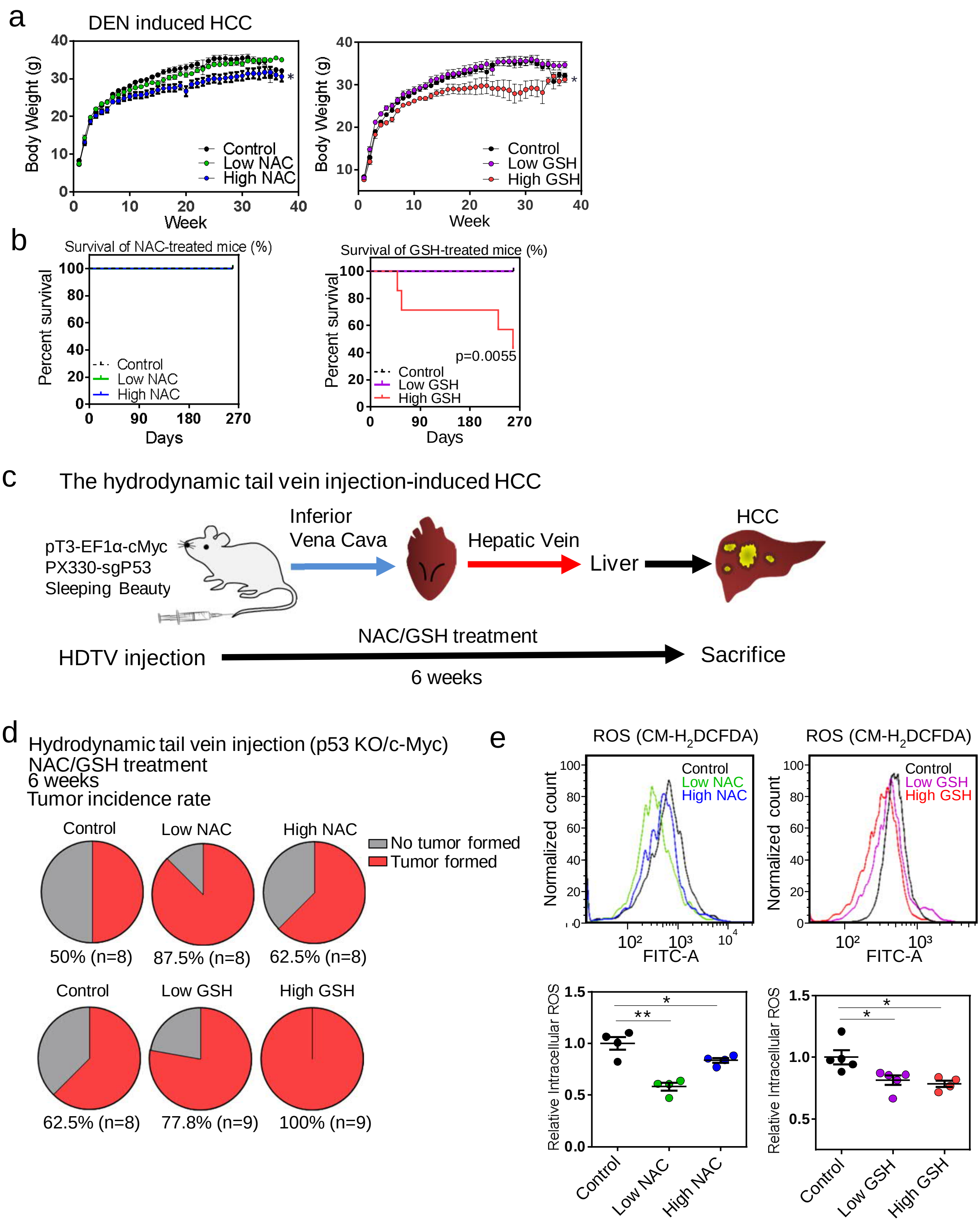

Supplementary Figure 2

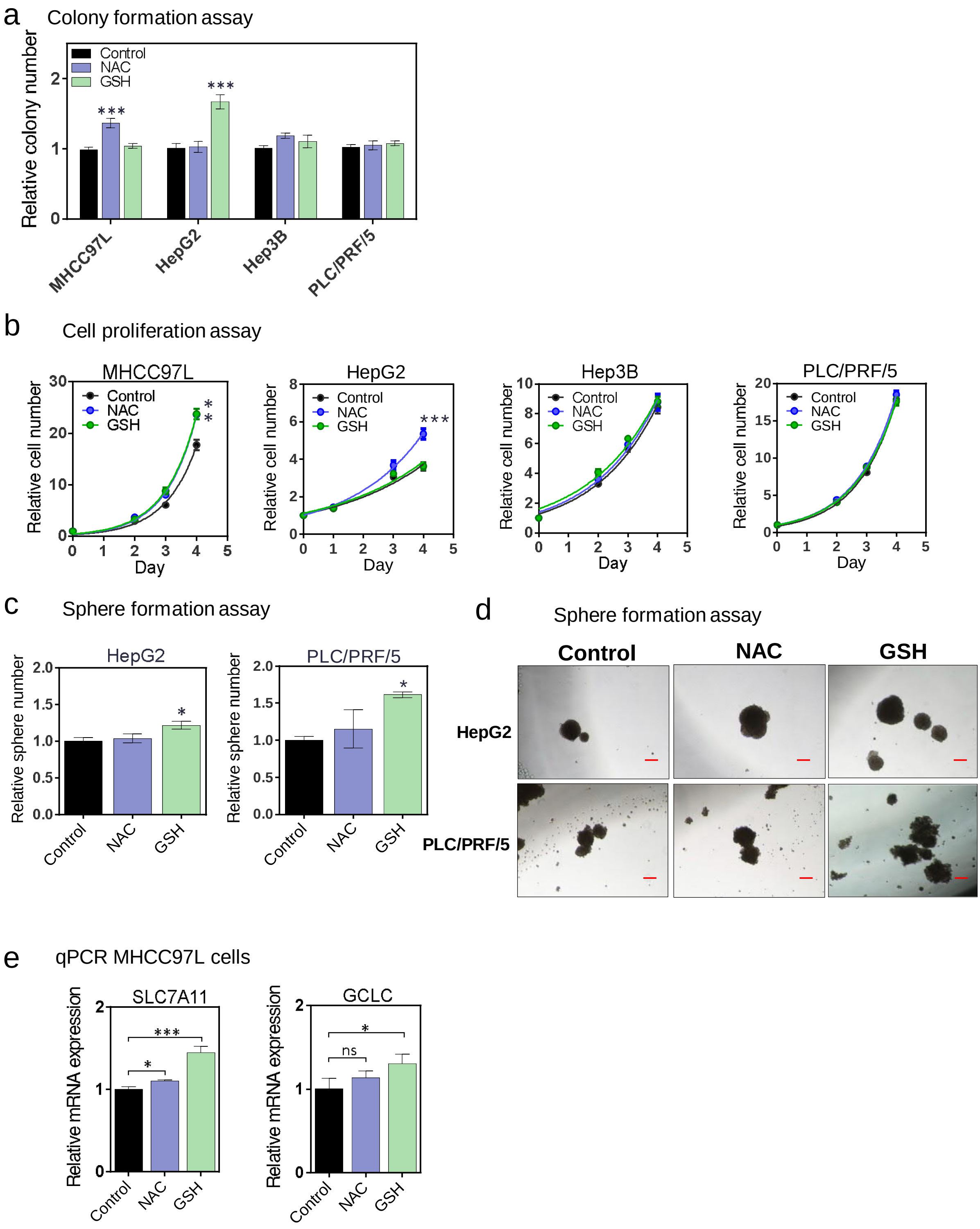

Supplementary Figure 3

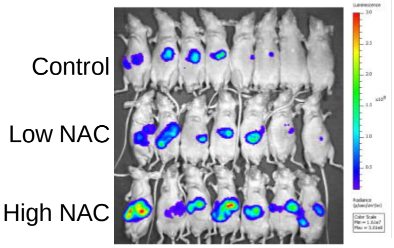

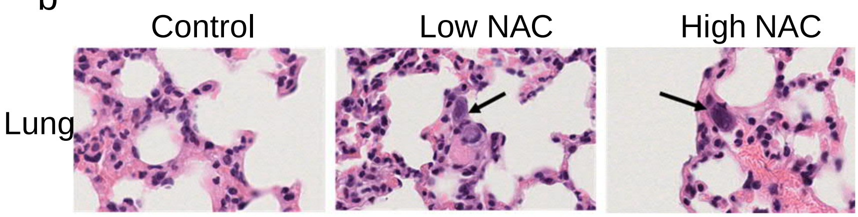

The metastatic foci		
Control	Low NAC	High NAC
0.23 (0-1)	0.37 (0-1.8)	0.22 (0-1.1)

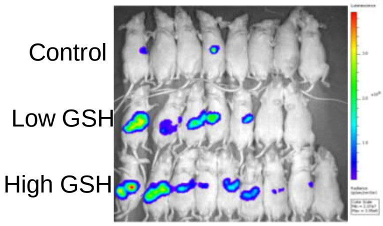

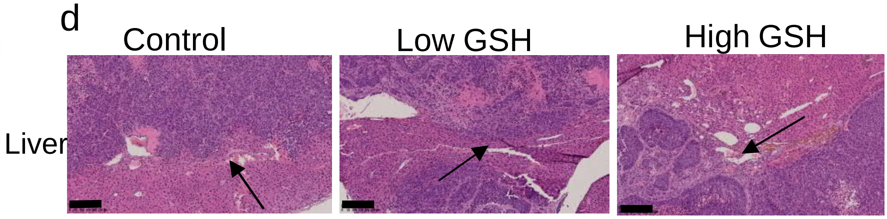

e

Tumor incidence rate

MHCC97L cells	Number of cells injected	Control	Low NAC	High NAC
Frequency	2,000	4/8	6/8	4/8
	20,000	4/8	6/8	7/8
	200,000	7/8	8/8	8/8

Tumor incidence rate

MHCC97L cells	Number of cells injected	Control	Low GSH	High GSH
Frequency	2,000	4/8	5/8	7/8
	20,000	6/8	7/8	8/8
	200,000	7/8	7/8	8/8

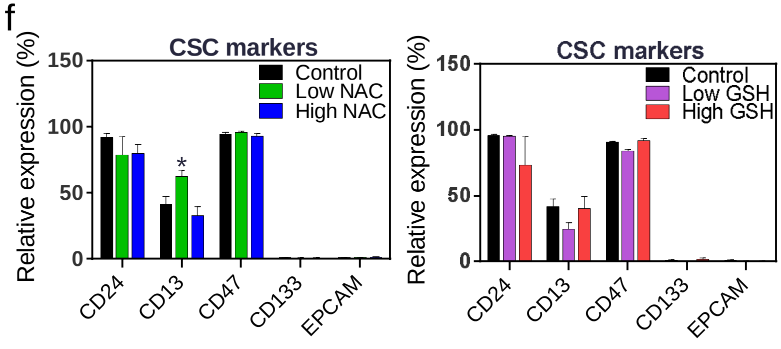

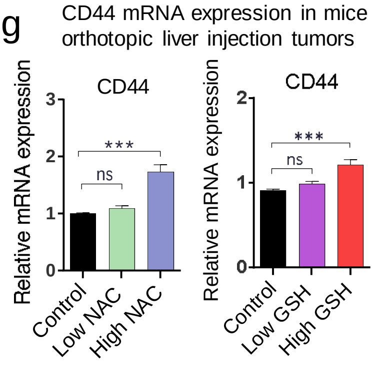

Supplementary Figure 4

a

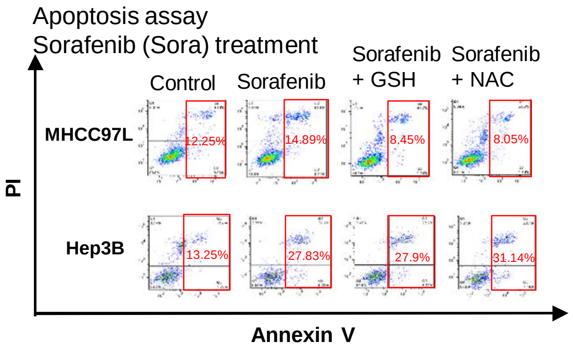

b

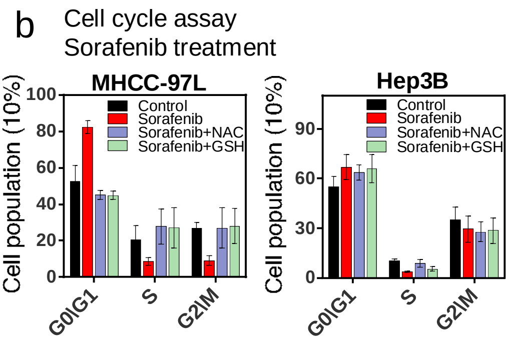

c

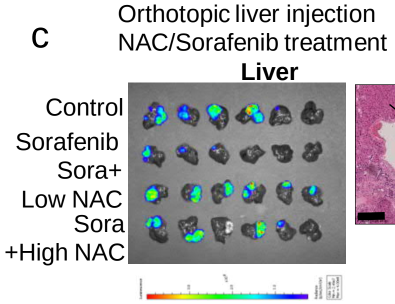

d

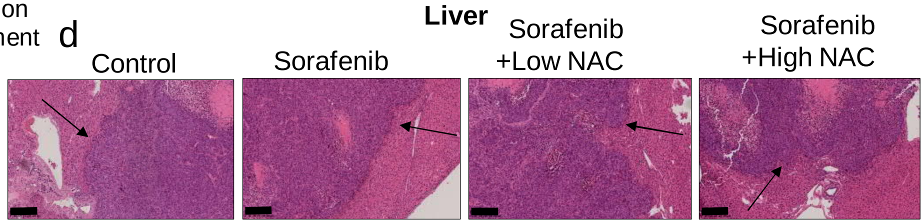

Lung

The metastatic foci in lung			
Control	Sorafenib	Sorafenib +Low NAC	Sorafenib +High NAC
0.03(0-0.1)	0	0.43* (0.2-0.7)	0.77 (0-2.1)

e

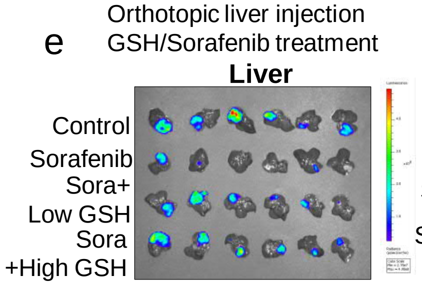

f

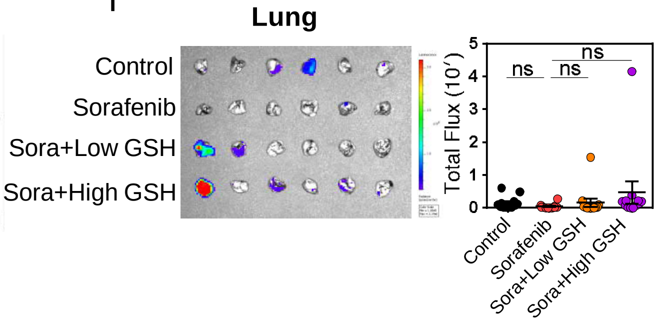

g

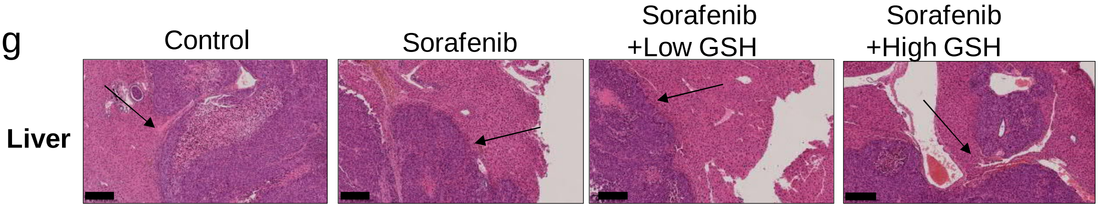

Lung

The metastatic foci in lung			
Control	Sorafenib	Sorafenib +Low NAC	Sorafenib +High NAC
0.03(0-0.1)	0	0.03 (0-0.1)	1.87 (0-0.9)

Supplementary Figure 5

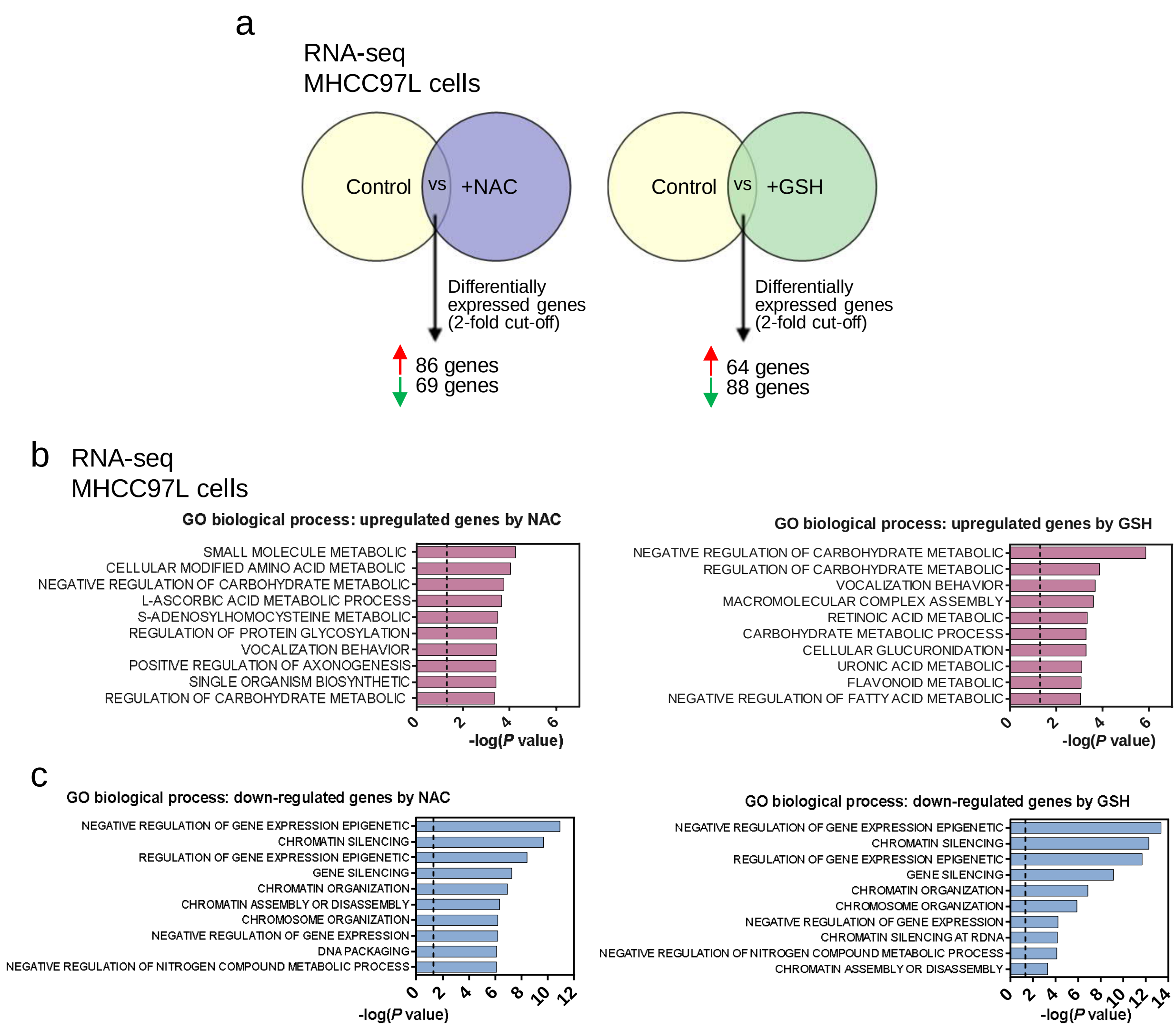

Supplementary Figure 6

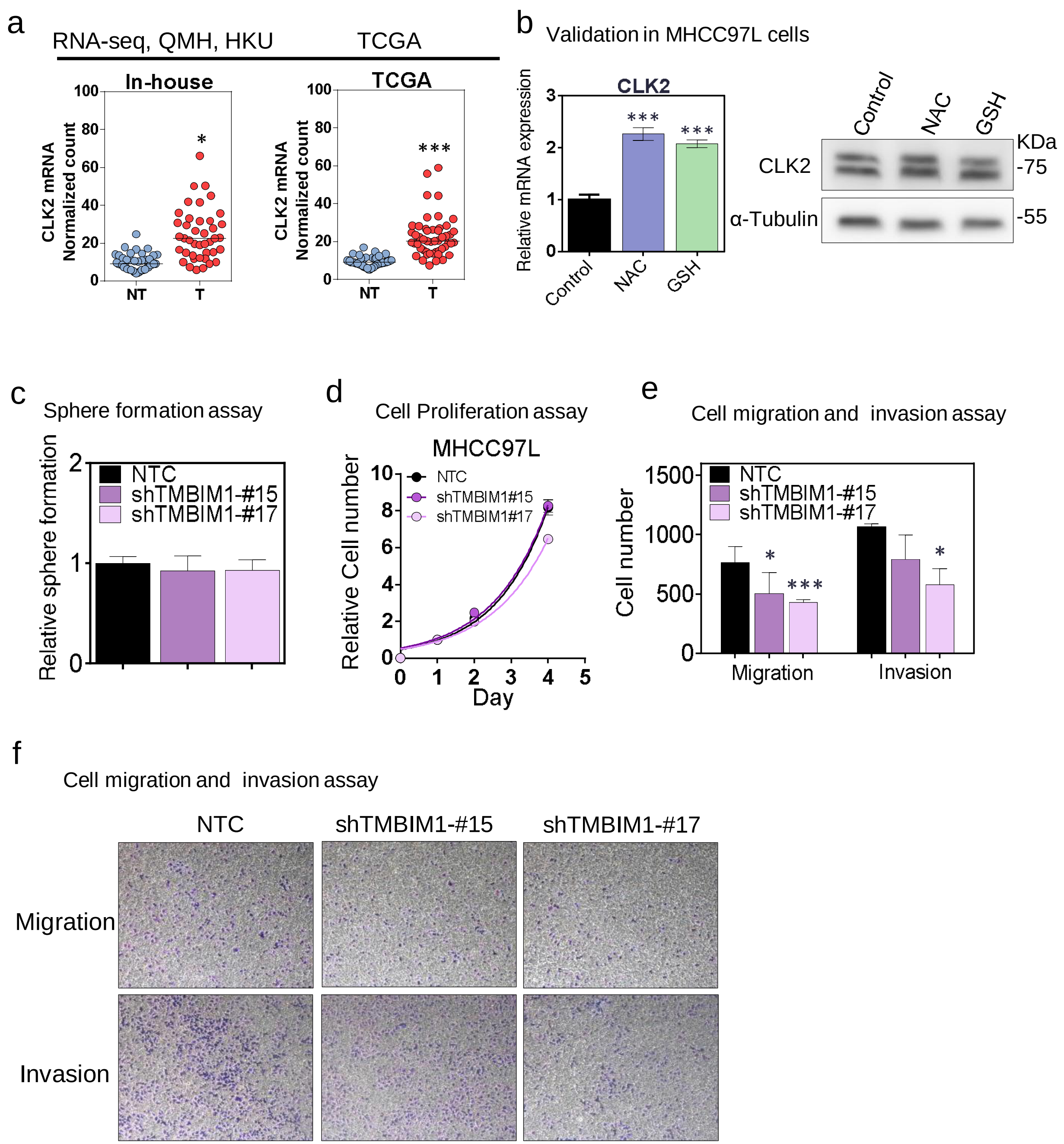

Supplementary Figure 7

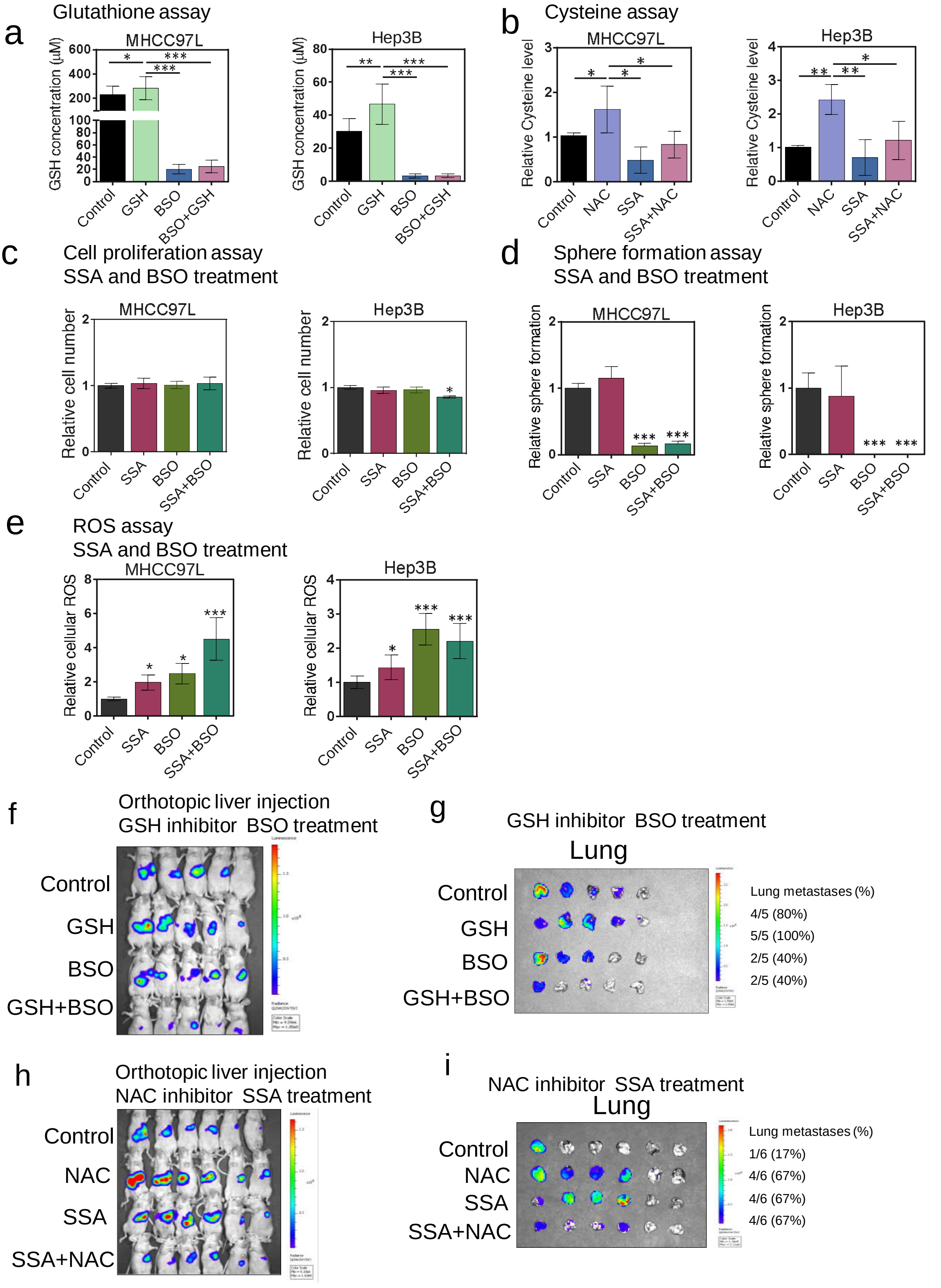

Supplementary Figure 8

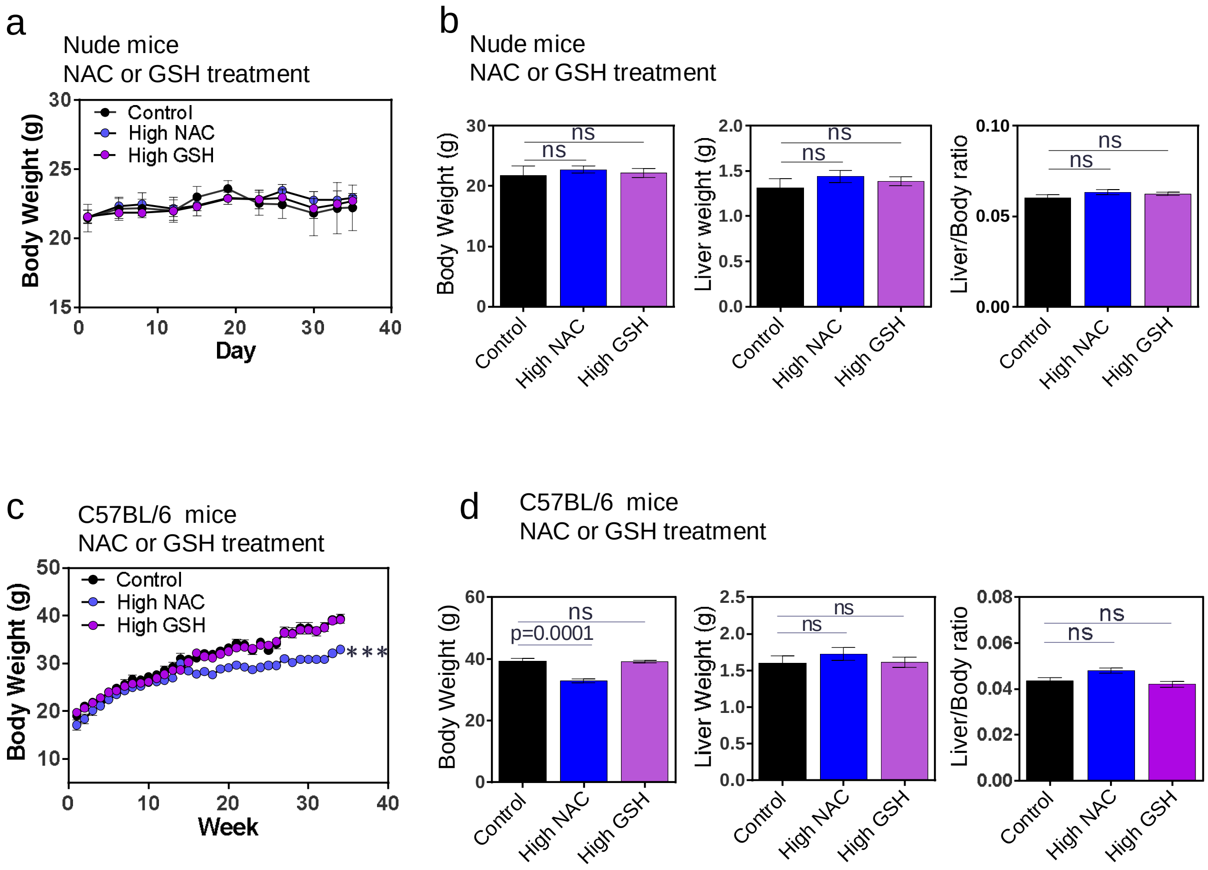

Supplementary Figure 9

Test Results:

DNA Marker	MHCC97L[4]	MHCC97L (L-171218744P)
AMEL	X, Y	X, Y
CSF1PO	11, 13	11, 13
D13S317	8	8
D16S539	12	12
D5S818	12,13	12,13
D7S820	10	10
TH01	9	9
TPOX	8	8
vWA	14	14
D18S51	--	13, 22
D21S11	--	31.2
D3S1358	--	15, 16
D8S1179	--	12, 13
FGA	--	21, 24
Penta D	--	8, 9
Penta E	--	11, 17
**Number of shared alleles**		12
**Total number of alleles in the reference profile**		12
**Percent match**		100%

Comment: The tested cell line has 100% match with MHCC97L. Therefore, they are considered to be related from a common ancestry.

Figure S9. STR authentication of MHCC-97L cells.

Supplementary Figure 10

a

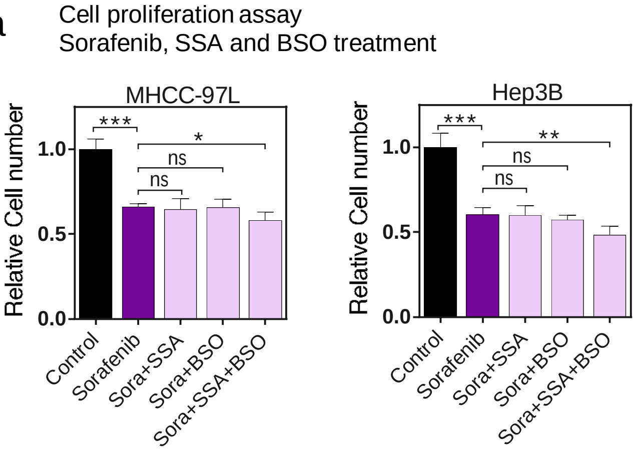

Supplement: Supplementary file 1 — Additional file 1: Fig S1. (a) The body weight changes upon NAC and GSH treatment in DEN-induced HCC mouse model. Data are expressed as mean ± SD from three independent experiments. (b) Survival rates of mice in NAC- and GSH-treated group when compared with control. (c) Hydrodynamic tail vein injection (HDTVi) (p53 KO/c-Myc) induced spontaneously tumorigenesis. (d) Tumor incidence of HDTVi (p53 KO/c-Myc) tumors in the presence of NAC treatment (upper panel) or GSH treatment (lower panel). ‘n’ represents the number of mice in each group. The tumor incidence was defined by the numbers of liver tumors > 1 mm in diameter. (e) Representative flow cytometry histogram (upper) and quantification (lower) of ROS levels in control, low-dose and high-dose NAC groups (left panel), and low- and high-dose GSH groups (right panel). Data are expressed as mean ± SD from three independent experiments. *P < 0.05, ** P < 0.01, and ***P < 0.001 vs. control; one-way ANOVA followed by Dunnett comparison test. Figure S2. (a) Colony formation assay in multiple HCC cell lines (MHCC7L, PLC/PRF/5, Hep3B and HepG2) with addition of NAC (1 mM), GSH (100 μM) and vehicle control. (b) Cell proliferation assay in MHCC-97L, HepG2, Hep3B and PLC/PRF/5 cells with treatment of NAC and GSH and in vehicle controls. (c)-(d) Sphere formation assay in HepG2 and PLC/PRF/5 cells with addition of NAC, GSH and control. (e) mRNA expression of SLC7A11 and GCLC by qPCR upon NAC and GSH treatment in MHCC97L cells. Data are expressed as mean ± SD from three independent experiments. *P < 0.05, **P < 0.01, ***P < 0.001 vs. NTC or control. one-way ANOVA followed by Dunnett comparison test. Figure S3. (a) Bioluminescent images of mice with orthotopic MHCC-97L cell injection and treated with control, low (60 mg/kg) and high dose (120 mg/kg) of NAC after tumor onset. (b) Representative H&E sections of lungs. Arrows indicate tumor cells found in lung tissues (lower). The frequencies of metastatic foci in the lungs were repre [file 13578_2021_731_MOESM1_ESM.pdf]
