# Supplementary material for: Antioxidant supplements promote tumor formation and growth and confer drug resistance in hepatocellular carcinoma by reducing intracellular ROS and induction of TMBIM1
Source: Cell Biosci. 2021 Dec 19;11:217. doi: 10.1186/s13578-021-00731-0 (PMC8684635; doi:10.1186/s13578-021-00731-0)
Supplement: Supplementary file 2 — Additional file 2: Table S1 Primer sequences used for qRT-PCR analysis. Table S2. Stable sh-knockdown sequences used in this study. [file 13578_2021_731_MOESM2_ESM.docx]

**Table S1** Primer sequences used for qRT-PCR analysis.

| Target gene |  | Sequence |
| --- | --- | --- |
| Human NRF2 | Forward | GGTTGCCCACATTCCCAAAT |
|  | Reverse | AGCAATGAAGACTGGGCTCT |
| Human GCLC | Forward | TTAGGCTGTCCTGGGTTCAC |
|  | Reverse | TCGCTCCTCCCGAGTTCTAT |
| Human SLC7A11 | Forward | ATGCAGTGGCAGTGACCTTT |
|  | Reverse | GGCAACAAAGATCGGAACTG |
| Human TMBIM1 | Forward | GACGGGCACCATTTCCAGTAT |
|  | Reverse | GGTGAAGTCCACCTTGGTCTGA |
| Human CLK2 | Forward | AATATTTTTACCGGGGTCGC |
|  | Reverse | AGCCGCTTAGCTGGTTCATA |
| Human CD24 | Forward | GCTCCTACCCACGCAGATTT |
|  | Reverse | GAGACCACGAAGAGACTGGC |
| Human CD13 | Forward | CTGTGAGCCAGTCTAGTTCCTGAT |
|  | Reverse | CATCGAGAGCTTCTGCTCATCT |
| Human CD44 | Forward | TGCCGCTTTGCAGGTGTAT |
|  | Reverse | GGCCTCCGTCCGAGAGA |
| Human CD47 | Forward | CAATCACGTAAGGGTCTCATAGG |
|  | Reverse | GATGGACTCCGATTTGGAGA |
| Human CD133 | Forward | TGGATGCAGAACTTGACAACGT |
|  | Reverse | ATACCTGCTACGACAGTCGTGGT |
| Human EPCAM | Forward | CCATGTGCTGGTGTGTGAAC |
|  | Reverse | ACGCGTTGTGATCTCCTTCT |
| Human SMO | Forward | TGGTCACTCCCCTTTGTCCTCAC |
|  | Reverse | GCACGGTATCGGTAGTTCTTGTAGC |
| Human NANOG | Forward | CCTGTGATTTGTGGGCCTG |
|  | Reverse | GACAGTCTCCGTGTGAGGCAT |
| Human CTNNB1 | Forward | GTGGGGCGCCCCAGGCACCA |
|  | Reverse | CTCCTTAATGTCACGCACGATTTC |
| Human SOX2 | Forward | AAATGGGAGGGGTGCAAAAGAGGAG |
|  | Reverse | CAGCTGTCATTTGCTGTGGGTGATG |
| Human C-MYC | Forward | CGTCCTCGGATTCTCTGCTC |
|  | Reverse | GCTGGTGCATTTTCGGTTGT |
| Human OCT4 | Forward | CTTGCTGCAGAAGTGGGTGGAGGAA |
|  | Reverse | CTGCAGTGTGGGTTTCGGGCA |
| Human NOTCH1 | Forward | TGAATGGCGGGAAGTGTGAA |
|  | Reverse | CACTTGTACTCCGTCAGCGT |
| HPRT | Forward | CTTTGCTGACCTGCTGGATT |
|  | Reverse | CTGCATTGTTTTGCCAGTGT |

**Table S2.** Stable sh-knockdown sequences used in this study.

| Target gene |  | Sequence |
| --- | --- | --- |
| shNRF2-#1 | Forward | CCGGTAAAGTGGCTGCTCAGAATCTCGAGATTCTGAGCAGCCACTTTATTTTTG |
|  | Reverse | AATTCAAAAATAAAGTGGCTGCTCAGAATCTCGAGATTCTGAGCAGCCACTTTA |
| shNRF2-#2 | Forward | CCGGGAGTTACAGTGTCTTAATACTCGAGTATTAAGACACTGTAACTCTTTTTG |
|  | Reverse | AATTCAAAAAGAGTTACAGTGTCTTAATACTCGAGTATTAAGACACTGTAACTC |
| shNRF2-#4 | Forward | CCGGCACCTTATATCTCGAAGTTCTCGAGAACTTCGAGATATAAGGTGTTTTTG |
|  | Reverse | AATTCAAAAACACCTTATATCTCGAAGTTCTCGAGAACTTCGAGATATAAGGTG |
| shTMBIM1 | Forward | CCGGCCGTTTCCCATGGAACATCATCTCGAGATGATGTTCCATGGGAAACGGTTTTTG |
| #15 | Reverse | AATTCAAAAACCGTTTCCCATGGAACATCATCTCGAGATGATGTTCCATGGGAAACGG |
| shTMBIM1 | Forward | CCGGCCGAAAGGTTTACTCCATCATCTCGAGATGATGGAGTAAACCTTTCGGTTTTTG |
| #17 | Reverse | AATTCAAAAACCGAAAGGTTTACTCCATCATCTCGAGATGATGGAGTAAACCTTTCGG |
| shNTC | Forward | CCGGTGGTTTACATGTTTTCTGACTCGAGTCAGAAAACATGTAAACCATTTTTG |
|  | Reverse | AATTCAAAAATGGTTTACATGTTTTCTGACTCGAGTCAGAAAACATGTAAACCA |
